# Different temporal requirements for *tartan* and *wingless* in the formation of contractile interfaces at compartmental boundaries

**DOI:** 10.1242/dev.200292

**Published:** 2022-10-31

**Authors:** Thomas E. Sharrock, Jenny Evans, Guy B. Blanchard, Bénédicte Sanson

**Affiliations:** Department of Physiology, Development and Neuroscience, University of Cambridge, Anatomy Building, Cambridge CB2 3DY, UK

**Keywords:** Actomyosin cortex, Tissue boundaries, Planar polarity, *Drosophila*, Gastrulation, Convergent extension, Germband extension, Parasegmental boundaries, Live imaging, Cell tracking, MS2-MCP system, LRR receptor

## Abstract

Compartmental boundaries physically separate developing tissues into distinct regions, which is fundamental for the organisation of the body plan in both insects and vertebrates. In many examples, this physical segregation is caused by a regulated increase in contractility of the actomyosin cortex at boundary cell-cell interfaces, a property important in developmental morphogenesis beyond compartmental boundary formation. We performed an unbiased screening approach to identify cell surface receptors required for actomyosin enrichment and polarisation at parasegmental boundaries (PSBs) in early *Drosophila* embryos, from the start of germband extension at gastrulation and throughout the germband extended stages (stages 6 to 11). First, we find that Tartan is required during germband extension for actomyosin enrichment at PSBs, confirming an earlier report. Next, by following in real time the dynamics of loss of boundary straightness in *tartan* mutant embryos compared with wild-type and *ftz* mutant embryos, we show that Tartan is required during germband extension but not beyond. We identify candidate genes that could take over from Tartan at PSBs and confirm that at germband extended stages, actomyosin enrichment at PSBs requires Wingless signalling.

## Teaser

**Summary:** An unbiased screen to identify cell surface receptors at a *Drosophila* model boundary reveals that actomyosin enrichment at parasegmental boundaries requires Tartan during early germband extension but Wingless signalling at later stages.

## INTRODUCTION

The mechanisms underlying the partitioning of groups of cells into immiscible compartments have fascinated scientists since the discovery of compartmental boundaries in *Drosophila* in the 1970s (Fagotto, 2020a). In many cases studied, this physical barrier is caused by a localised upregulation of actomyosin contractility at boundary cell-cell interfaces, found in both *Drosophila* and vertebrate models (Aliee et al., 2012; Calzolari et al., 2014; Canty et al., 2017; Landsberg et al., 2009; Monier et al., 2010). How this increase in cortex contractility is specified at boundary interfaces to create mechanical barriers remains only partially understood. Within homogeneous fields of epithelial cells, spatial regulation of transcription factors is key for the initiation of boundary formation (Dahmann et al., 2011; Monier et al., 2011). Downstream of these transcription factors, various cell surface receptors have been implicated in causing actomyosin enrichment at boundary interfaces. In vertebrates, for example at rhombomere boundaries in the hindbrain, the Ephrin/Eph receptors play a key role, but additional cell surface asymmetries have also been identified (Fagotto, 2020b; Pujades, 2020). In *Drosophila*, downstream receptors remained elusive for a long time, but recent work has started to identify specific cell surface asymmetries required for the formation of mechanical boundaries (Sharrock and Sanson, 2020; Wang and Dahmann, 2020).

Beyond its role in compartmental cell sorting, increase in cortical contractility at epithelial cell-cell junctions underlies many cell and tissue behaviours (Amack and Manning, 2012; Bielmeier et al., 2016; Bosveld et al., 2016; Collinet and Lecuit, 2021). In particular, convergent extension, whereby cells intercalate to elongate a tissue, was shown in *Drosophila* to require planar-polarised enrichment of the actomyosin cortex (Bertet et al., 2004; Collinet and Lecuit, 2021; Paré and Zallen, 2020; Zallen and Wieschaus, 2004). This planar polarisation is downstream of anteroposterior (AP) patterning, which generates the subdivision of the body axis by overlapping stripes of transcription factors encoded by the pair-rule genes (Bertet et al., 2004; Irvine and Wieschaus, 1994; Zallen and Wieschaus, 2004). In vertebrates, actomyosin planar polarisation has now been linked to convergent extension in several examples and, in contrast to *Drosophila* axis extension, is generally thought to be regulated by the planar cell polarity (PCP) pathway (Collinet and Lecuit, 2021; Nishimura et al., 2012; Paré and Zallen, 2020; Rozbicki et al., 2015; Shindo and Wallingford, 2014). It is not known, however, what upstream cues activate the PCP pathway to drive convergent extension in vertebrates and whether cell surface receptor asymmetries similar to those in *Drosophila* axis extension could contribute.

In this study, we have searched for cell surface receptors driving actomyosin enrichment at compartmental boundaries, using an unbiased screening approach. We focused on parasegmental boundaries (PSBs) that subdivide the germband in early *Drosophila* embryos. PSBs form during convergent extension of the germband during gastrulation, a process called germband extension (GBE). We had shown previously that actomyosin enrichments form at PSBs in the course of GBE, progressively emerging from the tissue-wide actomyosin planar polarisation that is initiated at gastrulation (Tetley et al., 2016). Tissue-wide planar polarisation of actomyosin requires the LRR receptors Toll-2 (also known as 18w), Toll-6 and Toll-8 (also known as Tollo), which are expressed in overlapping stripes downstream of the pair-rule transcriptional network (Paré et al., 2014). However, removal of all three receptors is not sufficient to abolish actomyosin enrichment at PSBs, suggesting that additional receptor(s) are required (Paré et al., 2019, 2014). In a previous study (Tetley et al., 2016), modelling cell-cell interactions during GBE, we proposed that an additional receptor, expressed in a periodic, double-segment pattern, might be sufficient to confer polarisation of actomyosin at PSBs. We undertook a systematic screen based on this hypothesis, which we present here. A second question concerned the role of surface receptors at different developmental stages. Once axis extension is completed, actomyosin enrichments are maintained at PSBs during the extended germband stages (Monier et al., 2010). Whereas actomyosin enrichments during convergent extension (GBE, stages 7-8) require the pair-rule gene network, their maintenance at PSBs after completion of GBE (stages 9-11) requires Wingless signalling (Monier et al., 2010; Scarpa et al., 2018; Urbano et al., 2018). No cell surface receptors had yet been identified downstream of Wingless signalling, so we also addressed this as part of our screening approach.

From our screen we find that Tartan, another LRR receptor, is required for actomyosin enrichment at PSBs throughout axis extension. This provides an independent confirmation of earlier findings by Paré et al. (2019). By visualising transcription of parasegmental markers in combination with cell tracking in live embryos, we were able to follow how boundary straightness (a functional consequence of actomyosin enrichment at boundaries) evolves in the course of axis extension in wild-type and *tartan* mutant embryos and also in the pair-rule mutant *ftz* (we examined *ftz* because it is a known regulator of *tartan* expression; Chang et al., 1993). This analysis showed that *tartan* is required for specifying contractile interfaces at PSB during early GBE, but not beyond. Our unbiased screen identifies candidate genes that could take over from Tartan to specify planar-polarised mechanical interfaces at PSBs.

## RESULTS

### A screen to find cell surface receptors expressed asymmetrically at PSBs

**Fig. 1.**
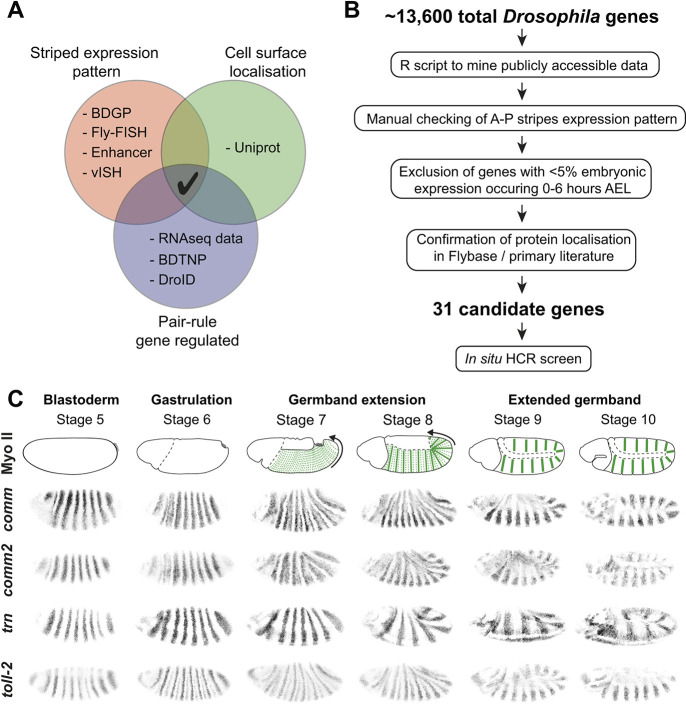
***In silico* screen to identify cell surface factors responsible for actomyosin enrichment at PSBs.** (A) Venn diagram illustrating the three criteria used to identify candidate cell surface factors in an *in silico* screen and including the main datasets that were mined. (B) Flow chart showing the successive steps taken to whittle down candidates. (C) *In situ* HCR images for key candidate genes found in the screen (inverted images of data in Fig. S1). *comm*, *comm2*, *tartan* and *Toll-2* are all expressed in clear AP stripes in early embryos. We distinguish two developmental periods for actomyosin enrichment at PSBs, GBE and extended germband stages. At the onset of GBE, actomyosin becomes planar polarised at every AP cell-cell interface, including PSB interfaces (dashed green lines, stage 7). As GBE progresses and cells intercalate (stage 8), actomyosin enrichment becomes more prominent at PSBs (solid green lines). Weaker actomyosin enrichment is also detectable at two intraparasegmental boundaries (dashed green lines) (Tetley et al., 2016). After completion of GBE, planar polarisation is lost except at PSBs (thick green lines, stages 9-10), where it is maintained throughout extended germband stages.

In previous work, we predicted that the expression of a single surface molecule within either even- or odd-numbered parasegments would constitute the minimal requirement for generating the missing molecular asymmetries at the parasegmental boundary during axis extension (Tetley et al., 2016). Based on this prediction, we performed an *in silico* screen to find genes meeting the following three criteria: (1) they should be expressed in stripes along the AP axis, (2) they should encode a protein that localises to the cell surface and (3) they should be regulated by the pair-rule gene network. Mining publicly available data for the 13,600 genes in the *Drosophila* genome, we identified 822 genes expressed in AP stripes, 5620 genes encoding proteins with a signal peptide and/or a predicted transmembrane domain and 3679 genes likely to be pair-rule regulated (Fig. 1A; see Materials and Methods). Following standardisation of the gene nomenclature using the unique FlyBase identifiers, we found 94 genes in common with these three datasets. Next, we applied manual quality controls to this initial list, whittling the number of candidates down to 31 genes (Fig. 1B; see Materials and Methods). Excluded genes were those (1) not showing an obvious striped pattern by eye in *in situ* libraries, (2) being likely to be expressed at very low levels in early embryos or (3) having a known localisation in the literature different from the cell surface (e.g. transcription factors) (see Materials and Methods for details).

**Fig. 2.**
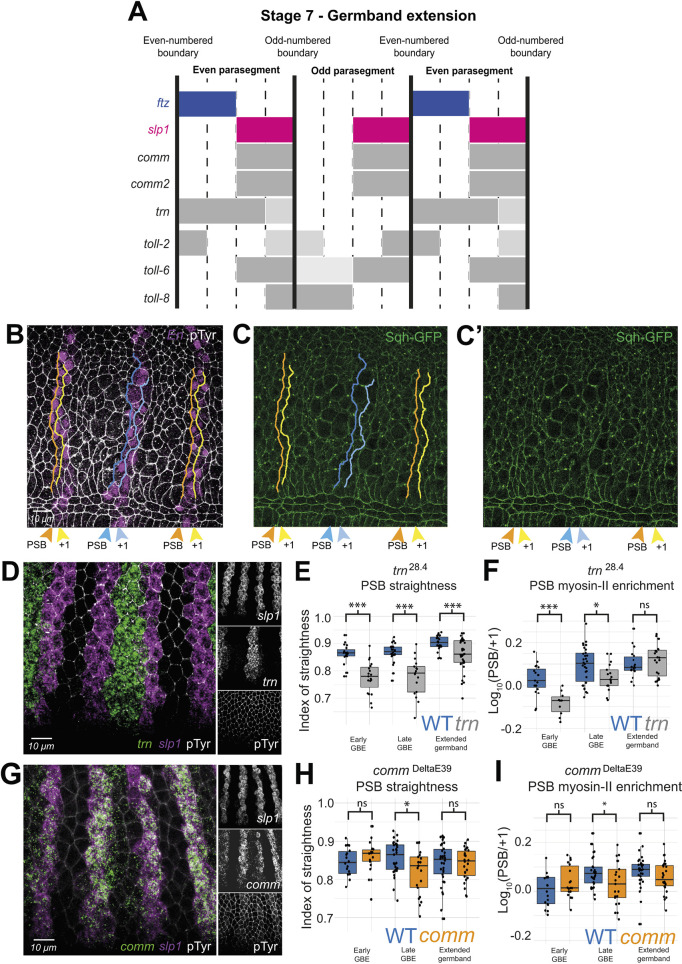
**Tartan is required for specification of contractile interfaces at PSB during GBE.** (A) Diagram based on *in situ* HCR images showing the expression patterns of *comm*, *comm2*, *tartan*, *Toll-2*, *Toll-6* and *Toll-8* in relation to boundary markers *ftz* and *slp1*, at the beginning of GBE (see all the stages in Fig. S3). Representative patterns are shown for even- and odd-numbered parasegments. Dark grey indicates high expression and light grey, lower expression. (B-C′) Example of quantifications in fixed embryos at stage 10. In B, lines of interest are drawn along the PSB and control +1 interfaces using Engrailed and pTyr staining, which label the PSBs and cell contours, respectively. Native fluorescence from *shq-GFP^KI^*, shown in C,C′, is then quantified along the lines. In this example, the orange and yellow lines have been used, whereas the blue lines could not be used because of the presence of cell divisions at the boundary. PSB lines are also used for measuring boundary straightness (see Materials and Methods). (D) *In situ* HCR of *tartan* expression during GBE*. tartan* is expressed within each even-numbered parasegments and abuts both odd- and even-numbered PSBs. (E) Index of straightness measures in wild type (WT) (*n*=74 PSBs, from 24 embryos) and *tartan* mutant embryos (*n*=74 PSBs, from 22 embryos). For this graph and all subsequent graphs, the box limits show the upper and lower quartiles and the bars extend to values within the upper quartiles plus 1.5× interquartile range; also the convention for *P*-values is: ns, not significant (*P*>0.05); *P<0.05; **P<0.01; ***P<0.001. (F) Myosin II enrichment measures at PSBs in WT (*n*=67 PSBs and 24 embryos) and *tartan* mutant embryos (*n*=50 PSBs and 21 embryos). (G) *In situ* HCR of *comm* expression during GBE. *comm* is expressed in a similar pattern to *sloppy paired* during GBE and abuts both odd- and even-numbered PSBs. (H) Index of straightness measures in WT (*n*=83 PSBs, from 26 embryos) and *comm* mutant embryos (*n*=65 PSBs for 27 embryos). (I) Myosin-II enrichment measures at PSBs in WT (*n*=83 PSBs, from 26 embryos) and *comm* mutant embryos (*n*=65 PSBs, from 27 embryos). ‘Early GBE’ corresponds to stage 7 embryos; ‘late GBE’, stage 8; ‘Extended germband’, stages 9 and 10.

Next, we used *in situ* hybridisation chain reaction (HCR) (Choi et al., 2018) to characterise the expression patterns of the 31 candidate genes during early embryogenesis relative to the parasegmental boundaries. HCR has several advantages over traditional *in situ* hybridisation: (1) because there is no enzymatic amplification, the signal is tightly localised within expressing cells, which helped identify the boundaries of expression with precision, (2) it enables the expression of several genes to be examined simultaneously and (3) it can be combined easily with antibody staining, which we used here to label the cell membranes to facilitate gene expression mapping relative to boundaries (see Fig. 2D,G). We focused on the period of embryogenesis from stage 5 (late cellularisation) to stage 10 (extended germband) (Fig. 1C). From 31 genes, we confirmed that 19 genes were expressed in AP stripes (Fig. S1), whereas 12 were not (Fig. S2) and were excluded from the candidate list. The 19 genes recovered included the three genes encoding the Toll-like receptors Toll-2, Toll-6 and Toll-8, already identified by Paré et al. (2014), thus validating our screening approach. The strength and type of striped patterns varied between the genes. Some were expressed in seven clear stripes at stages 5 to 7, indicating that they are likely under pair-rule gene network regulation: *Ama*, *Best1*, *comm*, *comm2*, *ImpL2*, *sca*, *Toll-2*, *Toll-6*, *Toll-8* and *tartan* (Fig. 1C, Fig. S1). Some genes also show clear expression in every parasegment at later stages 9-10, when the germband is extended, either doubling their periodicity from an initial expression in seven stripes (*comm*, *comm2*, *ImpL2*, *Toll-2*) or initiating expression in every parasegment (*dnt*, *drl*, *sli*) (Fig. S1). For the genes with the clearest striped expression, it was possible to check the position of the stripes relative to the parasegmental boundary markers *ftz* and *slp1.* We found that *Best1*, *blot*, *comm*, *comm2*, *dnt*, *ImpL2*, *Toll-6*, *Toll-8* and *tartan* mRNA expression borders the parasegment boundaries at some point between stages 5 and 10 (Fig. S3). We decided to focus further investigations on *comm*, *comm2* and *tartan*, as these genes were most clearly bordering the parasegmental boundaries and were also strongly expressed (Fig. 1C).

### A requirement for Tartan in actomyosin enrichment and boundary straightness at PSBs during GBE

Examining *tartan* expression patterns relative to PSBs revealed that, from all the 19 candidates, *tartan* was the gene matching our initial prediction the closest (Tetley et al., 2016). Like the Toll-like genes, *tartan* encodes an LRR receptor that localises at the plasma membrane (Chang et al., 1993). The protein localisation pattern visualised by antibody labelling matched the mRNA expression patterns, validating the use of HCR to map boundaries (Fig. S4A). We observed that from stage 5 to stage 7 *tartan* is expressed throughout the even-numbered parasegments and borders even-numbered PSBs at the anterior and odd-numbered PSBs at the posterior of its domains (Fig. 2A,D, Fig. S3). Note that we have summarised gene expression patterns in diagrams where representative parasegments are each divided into four domains (for example, see Fig. S3); although these four domains approximately correspond to the number of cells along AP at the beginning of GBE (3.7 cells on average; see Tetley et al., 2016), the cell number steadily increases with cell intercalation and then cell division (see Materials and Methods). *tartan* fulfilled our prediction of a single receptor expressed in either even or odd parasegments and bordering every PSB. However, the expression of *tartan* was not completely uniform across the even-numbered parasegments, being weaker towards the posterior of each stripe at stages 5 to 7 (see also Fig. S8). Towards the end of GBE (stage 8), the posterior border of *tartan* expression retracted away from the odd-numbered PSBs (Fig. S3). This is similar to the dynamics of expression of the pair-rule gene *ftz*, which is known to activate *tartan* expression (Chang et al., 1993). Note that from stage 8 onwards, the uniform expression of *tartan* started breaking down along the dorsoventral (DV) axis and the PSBs were bordered only intermittently (Fig. S1).

Next, we quantified both Myosin II enrichment and boundary straightness in a *tartan* null mutant (*trn^28.4^*), as a time course from stage 7 to stage 10. To measure Myosin II intensities, we used native fluorescence from Sqh-GFP^KI^, a knock-in reporter for Myosin II Regulatory Light Chain (Proag et al., 2019). Quantifications were performed in fixed embryos by comparing the cell-cell interfaces at PSBs to control interfaces one cell diameter posteriorly (called +1 interfaces) (see Fig. 2B-C′ for an example and Materials and Methods). The analysis showed a clear requirement for *tartan* for Myosin II enrichment at PSBs during GBE, this requirement being strong at GBE onset and subsequently diminishing, with no contribution during extended germband stages (Fig. 2F). PSB straightness quantifications followed a similar trend (Fig. 2E). This matched remarkably well the dynamics of expression of *tartan* mapped by HCR, suggesting that the specification of contractile cell-cell interfaces results from an immediate read-out of Tartan receptor asymmetries (Fig. S1). We conclude that our unbiased screening approach identifies a clear requirement for the LRR receptor Tartan to form actomyosin-enriched interfaces at PSBs, confirming a previous report (Paré et al., 2019). Our quantifications in fixed embryos further suggest that Tartan is required during early GBE, and that other cell surface receptors may be required later in development.

### Evaluating a possible requirement for Commissureless in boundary formation

Next, we considered the candidate genes *comm* and *comm2*, as they are expressed in clear stripes bordering PSBs during GBE (Figs 1C, 2A)*. comm* and *comm2* are duplicated genes located next to each other in the genome and exhibit identical expression patterns, which are markedly different from those of *tartan* (Fig. S1). At stages 5 and 6, their expression in seven stripes did not border any PSB and instead straddled the even-numbered PSBs. From stage 7 onwards, *comm* and *comm2* expression doubled in periodicity and the expression became localised to the second half of every parasegment, matching *slp1* expression to border the anterior side of each PSB (Fig. 2A,G). *comm* encodes a short transmembrane protein that does not localise to the cell surface but regulates the cell surface localisation of the receptor Robo and possibly other receptors (Ing et al., 2007; Keleman et al., 2002). Comm protein was detected in puncta, consistent with its known endoplasmic reticulum/Golgi localisation and formed a striped pattern that matched the RNA expression (Fig. S4B). Comm2 has not been characterised but its amino-acid sequence presents homology with Comm in key domains (Justice et al., 2017). We quantified both Myosin II enrichment and boundary straightness in a *comm* null mutant (*comm^Δe39^*) (Fig. 2H,I). We found a small but significant difference in Myosin II enrichment and boundary straightness late GBE (stage 8). This suggests that Comm, and perhaps its homologue Comm2, may have a role in boundary formation after Tartan.

### Evaluating a possible requirement for Toll-2 at PSBs during extended germband stages

**Fig. 3.**
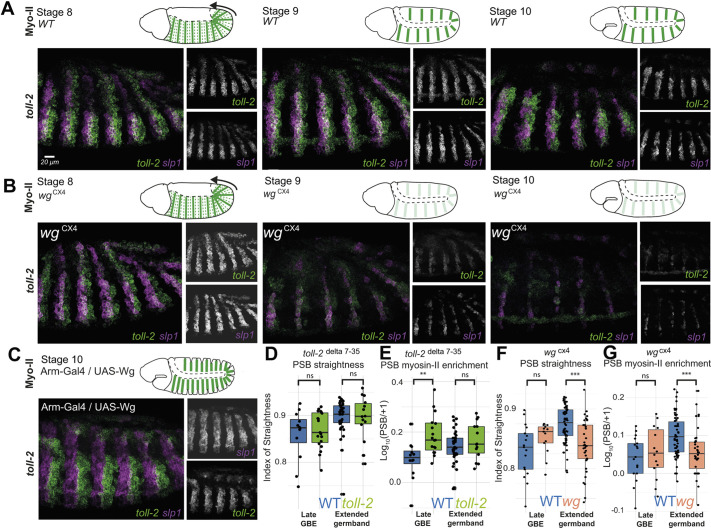
**Testing a requirement for *Toll-2* at PSBs during extended germband stages.** (A) Expression patterns of *Toll-2* and the PSB marker *slp1* in stage 8-10 wild-type (WT) embryos. Note that Toll-2 straddles the *slp* expression domain and thus the PSBs (merged signal in white). (B) Expression patterns of *Toll-2* and *slp1* in *wingless* mutant embryos. (C) Expression patterns of *Toll-2* and *slp1* in embryos overexpressing *wingless*. (D) Index of straightness measures in WT (*n*=49 PSBs, from 18 embryos) and *Toll-2* mutant embryos (*n*=33 PSBs, from 11 embryos). (E) Myosin II enrichment measures at PSBs in WT (*n*=49 PSBs, from 18 embryos) and *Toll-2* mutant embryos (*n*=33 PSBs, from 11 embryos). Note that the ratio PSB/+1 increases significantly at late GBE in the *Toll-2* mutant, consistent with a loss of actomyosin enrichment at +1 interfaces in this mutant, as described previously (Pare et al., 2019; Lavalou et al., 2021). (F) Index of straightness measures in WT (*n*=83 PSBs, from 25 embryos) and *wg* mutant embryos (*n*=50 PSBs, from 14 embryos). (G) Myosin II enrichment measures at PSBs in WT (*n*=83 PSBs, from 25 embryos) and *wg* mutant embryos (*n*=50 PSBs, from 14 embryos). Late GBE corresponds to stage 8 embryos; extended germband, stages 9 and 10.

In addition to looking at GBE, we also wanted to assess PSB function at extended germband stages (stages 9-11). At these stages, actomyosin enrichments initiated during GBE at PSBs are maintained by Wingless signalling (Monier et al., 2010). In *wingless* mutants, both actomyosin enrichment and boundary straightness are lost at PSBs, as is the elevated tension along the cell-cell interfaces, shown by laser-ablation experiments (Monier et al., 2010; Scarpa et al., 2018; Urbano et al., 2018). We reasoned that any cell surface receptor contributing to maintaining actomyosin enrichments at PSBs must be under Wingless signalling regulation. We therefore performed HCR in a *wingless* null mutant (*wg^CX4^*), for the candidate genes expressed in stripes at extended germband stages (*Best1*, *comm*, *comm2*, *dnt*, *drl*, *sli*) as well as for *tartan* and the Toll-like receptor genes (Fig. S5). Of these 11 genes, only *Toll-2* lost expression in *wg^CX4^* embryos compared with wild type (Fig. 3A,B, Fig. S5). To confirm regulation by Wingless signalling, we examined *Toll-2* expression (alongside the other Toll-like receptor genes and *tartan* as controls) in embryos ubiquitously expressing Wingless (*armGal4/UASwg*). Again, *Toll-2* was the only gene responding robustly to Wingless signalling, its expression broadening towards the posterior to reach the anteriorly broadened expression of the *slp1* domain (Fig. 3C, Fig. S5). The broadening of *Toll-2* expression is similar to the broadening in *engrailed* expression (a known target of Wg) in *armGal4/UASwg* embryos (Larsen et al., 2008; Scarpa et al., 2018; Urbano et al., 2018), confirming that Wg signalling regulates *Toll-2* expression.

Regulation of *Toll-2* by Wingless signalling could contribute to the maintenance of actomyosin enrichment at PSBs. *Toll-2* expression, however, does not border the PSBs at extended germband stages, but rather straddles it, similarly to its pattern during GBE (Fig. 3A, Fig. S3). To address a possible role of Toll-2 at PSBs, we quantified Myosin II enrichment and boundary straightness in a *Toll-2* null mutant (*toll-2^delta7-35^*) (Fig. 3D,E). Consistent with previous reports (Lavalou et al., 2021; Paré et al., 2019, 2014), we did not detect a loss of actomyosin enrichment or boundary straightness at PSBs in *Toll-2* mutants during GBE (Fig. 3D,E). Despite the regulation of *Toll-2* by Wingless signalling, we also could not detect a contribution of *Toll-2* at extended germband stages (Fig. 3D,E). As a positive control, we quantified actomyosin enrichment and boundary straightness in *wg* null mutants and found, as expected, a very significant decrease at extended germband stages (and not during GBE) (Fig. 3F,G). This confirms that our quantifications of Sqh-GFP native fluorescence are comparable to our prior quantifications using a P-Sqh antibody (Monier et al., 2010; Urbano et al., 2018). Together, these results suggest that regulation of *Toll-2* by Wingless signalling does not explain the maintenance of actomyosin enrichments at PSBs at germband extended stages.

### Tools to monitor boundary activity during GBE in live embryos

Our analysis in fixed embryos suggests that the requirement for Tartan is limited to early convergent extension, matching closely its window of expression at PSBs. This is intriguing because it suggests that the specification of contractile interfaces is a rapid and short-lived response to the asymmetric expression of Tartan at boundaries. In order to analyse more precisely the dynamics of requirement for *tartan*, we developed tools to monitor boundary mechanical properties during axis extension. In previous work, we had quantified Myosin II polarity in live embryos using Sqh-GFP (a reporter for Myosin II Regulatory Light Chain), while tracking cells with the cell membrane marker Gap43-Cherry (Tetley et al., 2016). Here, we developed boundary straightness measurements in real time as a functional assay for boundary mechanical properties and a proxy for actomyosin enrichment.

To follow the dynamics of boundary straightness in live embryos, we took advantage of the MS2-MCP system implemented in *Drosophila* embryos (Garcia et al., 2013), to label in real time the transcription of the parasegmental boundary marker *engrailed* (Fig. S6A). The reason for using a transcriptional read-out rather than a protein reporter is that we found that tagged proteins of segmental markers, constructed by others or ourselves, do not give a fluorescent signal strong enough for tracking parasegments in live embryos. One exception is *eve-YFP*, which we used previously, but this has the limitation of labelling only alternate parasegmental boundaries (Tetley et al., 2016). We fused a 2099 bp region upstream of the *engrailed* promoter, the VT15159 enhancer, to an MS2 reporter containing 24 MS2 stem loops and *lacZ*, generating the construct EnVT15159-MS2 (Fig. S6B; see Materials and Methods). We checked by HCR that *lacZ* expression from this construct recapitulates the endogenous pattern of *engrailed* expression during axis extension (Fig. S6C,D). The only difference was brighter Ftz-positive stripes (marking even-numbered parasegments), which might be a consequence of the known delay in transcription initiation of *engrailed* in odd-numbered parasegments compared with even-numbered ones (DiNardo et al., 1988).

**Fig. 4.**
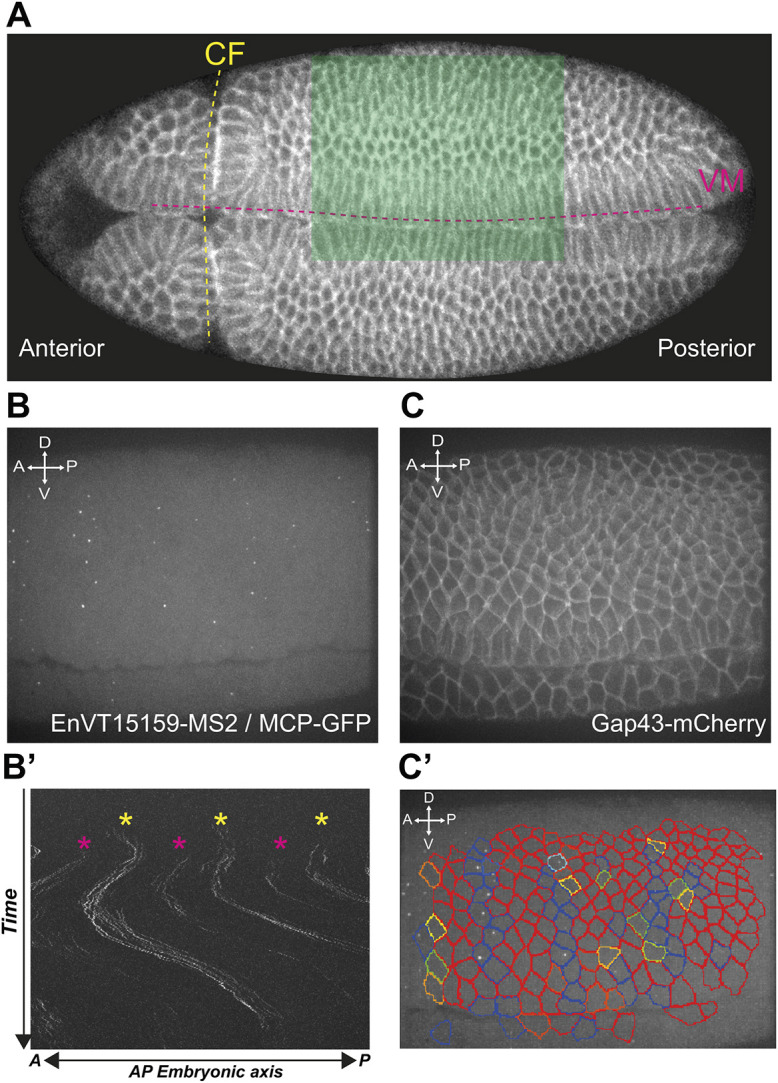
**Tracking PSBs in live *Drosophila* embryos.** (A) Spinning disc confocal image of a live gastrulating *Drosophila* embryo expressing Gap43-mCherry. The cephalic furrow (CF) and ventral midline (VM) are annotated with dashed lines. The ventrolateral region shaded green is the approximate area imaged via spinning disc confocal microscopy. (B,C) Maximum intensity projections of a single time frame from an embryo expressing the transgenes EnVT15159-MS2, MCP-GFP and Gap43-mCherry. MCP-GFP binds the MS2 loops on nascent mRNA transcribed from the *engrailed* enhancer EnVT15159 and form spatially localised fluorescence within nuclei that resemble dots. Gap43-mCherry protein localises to cell membranes. (B′) Kymograph showing the distribution of transcriptional dots along the embryonic axis during GBE. Magenta asterisks indicate stripes that coincide with odd-numbered PSBs, yellow asterisks indicate the brighter stripes that coincide with even-numbered PSBs. (C′) Movie frame showing cell segmentation based on the Gap43-mCherry signal and boundary cell tracking based on the MCP-GFP dot signal. Assigning the dots to cells reveals the position of Engrailed-expressing cells. A, anterior; D, dorsal; P, posterior; V, ventral.

Next, we visualised transcription from EnVT15159-MS2 by co-expressing MCP-GFP, which binds to the 24 MS2 stem loops in nascent transcripts and gives rise to fluorescent dots corresponding to *engrailed* transcription in the nuclei of live embryos (Fig. 4B, Fig. S6A). A kymograph of the dots revealed that, as for *lacZ* expression from the same reporter (Fig. S6C,D), reported *engrailed* transcription is brighter in alternate parasegments (Fig. 4B′). This was useful as it gave us a means to distinguish even-numbered from odd-numbered parasegments. To associate *engrailed* transcriptional dots with given cells, we used Gap43-mCherry, as previously, to label cell membranes and track cell positions (Fig. 4C) (Tetley et al., 2016). We developed additional computational methods to track the transcriptional dots in order to identify *engrailed*-positive cells (Fig. 4C′) and thereby the cell-cell interfaces of the parasegmental boundaries (Fig. S7; see Materials and Methods). As shown below, we found that the parasegmental boundaries identified by these methods are, as expected, significantly straighter than control (non-boundary) interfaces throughout axis extension, thus validating the use of a reporter of *engrailed* transcription to identify parasegmental boundaries in live embryos.

### Mapping Tartan's temporal requirement for parasegmental boundary straightness during axis extension

**Fig. 5.**
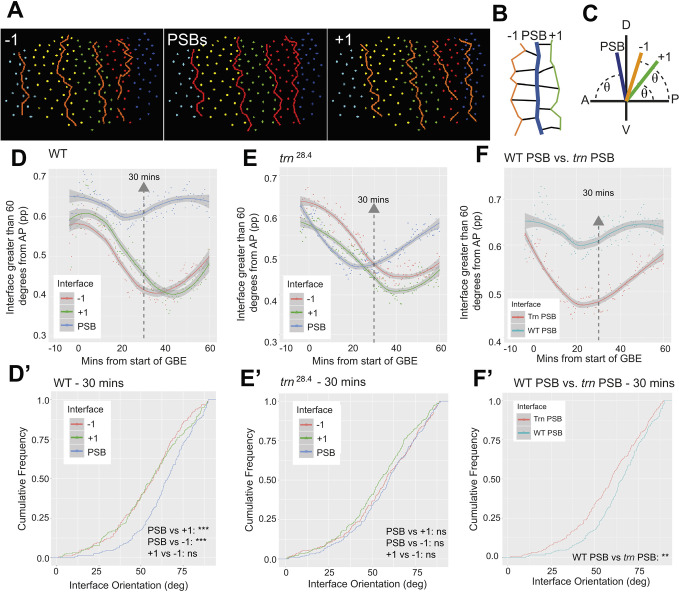
**Live imaging reveals Tartan is required for PSB straightness throughout GBE.** (A) In tracked movies, cell centroids coloured by parasegment identity. PSBs, −1 and +1 interfaces are overlayed to show their position relative to one another. (B) Diagram showing the relative position of −1, PSB and +1 interfaces. −1 and +1 interfaces correspond to the column of AP interfaces one cell diameter to the anterior and to the posterior of PSBs, respectively. (C) Diagram showing how the orientation of cell interfaces that make up each column is calculated. θ represents the measured angle between the embryonic AP axis and each cell interface. Angles of each cell interface that make up a column are measured automatically in our tracking software. (D,E) Plots showing the proportion of interfaces at PSB and control −1 and +1 interfaces that are greater than 60° from the AP axis, in the course of GBE, for three wild-type (D) and three *tartan* mutant (E) embryos. A LOESS curve (span 0.75) has been fitted to the data, with the shaded ribbon indicating a 95% confidence interval. (D′,E′) Statistical comparisons at time point 30 min with a Kolmogorov–Smirnov non-parametric test undertaken on the cumulative frequencies of interface angles. (F,F′) Subset of the curves shown in D and E to directly compare the PSB straightness in wild-type (WT) and *tartan* mutant embryos in the course of GBE, with statistics for time point 30 min in F′.

To compare parasegmental boundary straightness, we analysed three movies each of wild-type and *tartan* mutant embryos carrying the transgenes *EnVT15159-MS2*, *MCP-GFP* and Gap43-mCherry. Movies were acquired as before (Tetley et al., 2016) (see field of view in Fig. 4A), cells were segmented automatically based on the Gap43-mCherry signal, then segmentation was corrected manually (see Materials and Methods). Manual correction was important to recover enough cell-cell interfaces for the boundary straightness analysis. *engrailed* transcriptional dots from *EnVT15159-MS2/MCP-GFP* were tracked to locate the *engrailed* stripes and to find the parasegmental boundaries at the anterior border of each stripe (Fig. S7). We then compared the angle of cell-cell interfaces relative to the AP axis for parasegmental boundary interfaces and for control interfaces located one cell diameter posteriorly or anteriorly (+1 and −1 interfaces, respectively) (Fig. 5A-C). In wild-type embryos, cell-cell interfaces were relatively straight at the beginning of axis extension, with 55-65% of PSB and control interfaces having an angle greater than 60° relative to the AP axis (Fig. 5D). This initial interface straightness is caused by the invaginating mesoderm pulling on the ventral border of the ectoderm around the time axis extension starts (Butler et al., 2009; Lye et al., 2015). Once the mesoderm has invaginated, the control interfaces lost their alignment as cells intercalated during axis extension. In contrast, parasegmental boundaries remained aligned throughout axis extension, with 60% of boundary interfaces having an angle greater than 60° relative to the AP axis (Fig. 5D,D′). These trends were remarkably similar to our previous measurements for even-numbered PSBs identified using Eve-YFP (see figure 2K,L in Tetley et al., 2016), validating our new method to identify boundaries, and confirming that parasegmental boundaries behave as mechanical boundaries during *Drosophila* GBE.

In *tartan* mutants, the control interfaces anterior and posterior to the PSBs (−1 and +1) showed the same behaviour as in wild-type embryos: they were initially aligned at the start of GBE by the mesoderm pull, then lost their alignment as cells start intercalating. Strikingly, parasegmental boundaries in *tartan* mutants, in contrast with wild-type ones, lost their alignment even more quickly than control interfaces (Fig. 5E,E′). This suggests a complete absence of actomyosin enrichment at PSBs in *tartan* mutants at the start of axis extension. Moreover, direct comparison between *tartan* and wild type demonstrated that PSBs in *tartan* null mutants are less straight throughout axis extension (Fig. 5F,F′). The straightness evolution over time in *tartan* mutants was consistent with our time-course of actomyosin enrichment and boundary straightness in fixed embryos (Fig. 2C,D). At 30 min into GBE, the straightness of *tartan* and control interfaces were indistinguishable (Fig. 5E); from this point on, the PSB straightness started to recover, diverging from control interfaces (Fig. 5E) and increasing towards wild type (Fig. 5E). This suggests that other receptor systems take over then to promote actomyosin enrichment at PSBs. It also suggests that boundary straightness is an immediate read-out of the molecular asymmetries present at a given period of development.

### Pair-rule regulation of *tartan* and its implication for boundary formation during GBE

Our quantifications in fixed and live *tartan* embryos suggest that Tartan is the only patterned receptor required for actomyosin enrichment and straightness of PSBs at the start of GBE. This early requirement is consistent with the expression of *tartan* being controlled along the AP axis by pair-rule genes. Indeed, *tartan* is reported to be regulated by Ftz (Chang et al., 1993). At stage 5, *ftz* is expressed throughout even-numbered parasegments, strongly at even-numbered PSBs and diminishing toward the odd-numbered PSBs. Because this tailing off appears to match the expression of *tartan* (Fig. S3), the simplest hypothesis was that Ftz is the sole regulator of *tartan* at both even- and odd-numbered PSBs, which we went on to test.

**Fig. 6.**
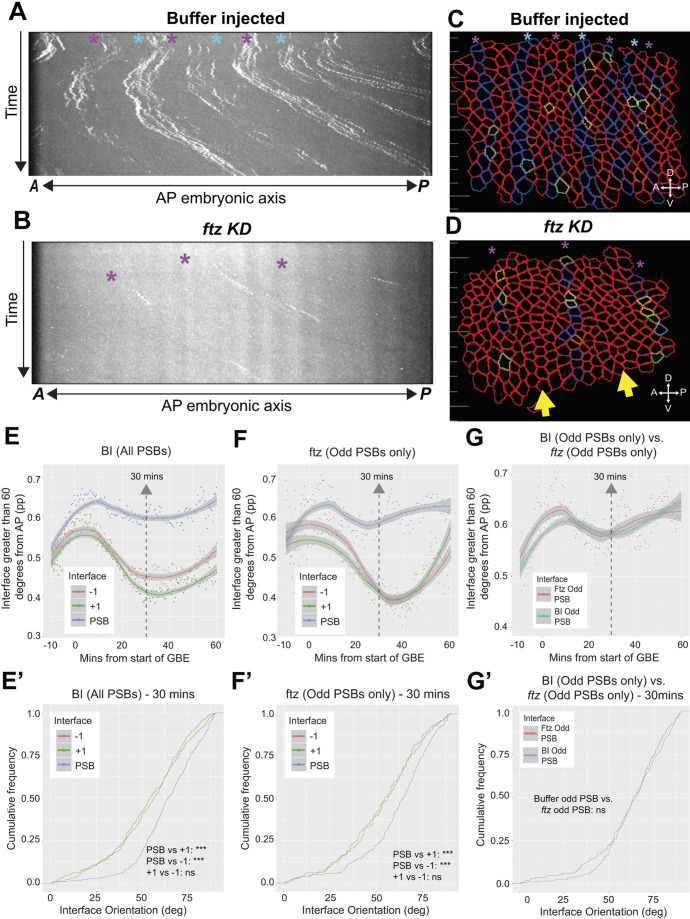
**Boundary tracking in live embryos reveals that odd-numbered PSBs in *ftz* mutant embryos retain their straightness during GBE.** (A,B) Kymographs showing the spatiotemporal distribution of transcriptional MCP-GFP dots marking the PSBs during GBE. Magenta asterisks indicate stripes that coincide with odd-numbered PSBs, blue asterisks indicate the brighter stripes that coincide with even-numbered PSBs. In A, buffer-injected embryos show the same pattern as wild-type embryos (compare with Fig. 4B′). In B, embryos injected with *ftz* dsRNA (*ftz* KD) have, as expected, lost the stronger stripes that coincide with even-numbered PSBs. (C,D) Movie frames showing examples of boundary tracking in a buffer-injected embryo (C) and a *ftz* KD embryo (D). Yellow arrows indicate the approximate position where even-numbered *engrailed*-positive stripes are missing in *ftz* KB embryos. (E,F) Straightness measurements in the course of GBE for four buffer-injected (BI) and four *ftz* KD embryos. Plots show the proportion of interfaces that are greater than 60° from the AP axis, for PSBs and +1 and −1 control interfaces. A LOESS curve (span 0.75) has been fitted to the data, with the shaded ribbon indicating a 95% confidence interval. Note that for *ftz* KD (F), only the odd-numbered PSBs and corresponding control interfaces could be tracked. (E′,F′) Statistical comparison for time point 30 min for the plots shown in E and F uses a Kolmogorov–Smirnov non-parametric test undertaken on the cumulative frequencies of interface angles. (G,G′) Subset of data from the curves shown in E and F to directly compare the straightness of odd-numbered PSBs in buffer-injected and *ftz* KD mutant embryos in the course of GBE, with statistics for time point 30 min in G′. A, anterior; D, dorsal; P, posterior; V, ventral.

We analysed boundary straightness in live *ftz* knockdown embryos, using the same methods as for *tartan* mutants. *ftz* dsRNA injection resulted in pair-rule cuticle phenotypes identical to those of *ftz* null mutants, showing that we have an efficient knockdown (Fig. S9D,E). We analysed three movies each of *ftz* dsRNA-injected and control buffer-injected embryos. *engrailed* transcriptional dots in buffer-injected embryos had brighter even-numbered stripes of *engrailed* dots, as in wild type (Fig. 6A,C). Straightness curves were similar to wild type, with PSBs consistently straighter than control interfaces throughout axis extension (Fig. 6E,E′). In the *ftz* knockdown, we expected the even-numbered stripes to be lost, because Ftz is required to activate *engrailed* transcription in even-numbered parasegments (Florence et al., 1997; Howard and Ingham, 1986). Consistent with this, we found that alternate stripes of *engrailed* transcriptional dots were gone, with weak stripes remaining, which we inferred were the odd-numbered stripes (Fig. 6B). Although weak, we were able to use those traces to track the odd-numbered PSBs in *ftz* knockdown embryos (Fig. 6D). If *ftz* is the sole regulator of *tartan* along the AP axis, the prediction is that odd-numbered PSBs should lose their straightness in *ftz* mutants, because *tartan* would be gone. This was not what we found: odd-numbered PSBs were clearly straighter than control interfaces in *ftz* RNAi embryos (Fig. 6F,F′) and they had the same straightness as in buffer-injected embryos when compared side-by-side (Fig. 6G,G′). We conclude that odd-numbered PSBs are fully functional in *ftz* mutant embryos.

Two possibilities could explain the above result: either there is enough *tartan* expression remaining at odd-numbered PSBs to support boundary formation, or other receptors, such as *Toll-like 2*, *6* or *8* rescue boundary formation via changes in their transcription patterns in the absence of *ftz*. To address this, we performed HCR in *ftz* null mutants to map the expression patterns of these four genes (and also *Best1*, *comm*, *comm2*, *dnt*, *drl* found in our screen) relative to both odd- and even-numbered PSBs (Fig. S8A,B). We found that, although the expression of most of these genes changed in the region where even-numbered PSBs had been lost, their expression was unchanged at odd-numbered PSBs. In particular, *tartan* was still expressed at odd-numbered PSBs in *ftz* mutants. Our quantification shows that although the expression of *tartan* bordering even-numbered PSBs was gone, as expected, its expression bordering odd-numbered boundaries was unchanged (Fig. S8C-F). We conclude that the simplest explanation for why odd-numbered PSBs still function normally in *ftz* mutants is that *tartan* expression is maintained there, presumably under other pair-rule regulatory inputs.

The above results suggest that *tartan* expression at even- and odd-numbered PSBs is regulated independently. We wondered whether this had a measurable impact on boundary function. To address this, we classified odd- and even-numbered PSBs using the stronger signal for *engrailed* transcriptional dots in even-numbered PSBs. In both wild type and buffer-injected controls, even-numbered PSBs were slightly straighter than odd-numbered ones throughout most of extension (Fig. S9B,C). This is consistent with an independent regulation of Tartan at both classes of PSBs. These differences, however, were subtle, and our statistical tests at a couple of time points were not significant (Fig. S9B′,C′). We also investigated the requirement for Tartan at either boundary and found that the loss of straightness at PSBs in *tartan* mutants was the same at odd- versus even-numbered PSBs (Fig. S9A,A′). We conclude that *tartan* is required for actomyosin enrichment at every PSB during early extension.

## DISCUSSION

One of our motivations for taking a systematic approach was to evaluate how many cell surface receptors are required for compartmental boundary formation during axis extension. We previously proposed (Tetley et al., 2016) that a single receptor expressed in either even- or odd-numbered stripes would be the minimal number. Tartan fits this single receptor hypothesis because it is expressed in even-numbered parasegments and is required for polarised contractility of interfaces at every parasegment boundary during axis extension (this study and Paré et al., 2019). What we additionally show here is that Tartan is required from the start of axis extension and that this requirement decreases as axis extension progresses, as shown by our quantifications over time in fixed (Fig. 2E,F) and live (Fig. 5E-F′) embryos. This suggests that other inputs take over to maintain actomyosin enrichment at PSBs during late axis extension and extended germband stages.

We have identified six other genes that encode cell surface receptors or regulators of cell surface receptors and that are differentially expressed at parasegment boundaries during axis extension: *Best1*, *blot*, *comm*, *comm2*, *dnt* and *ImpL2*. *comm* and *comm2* have the clearest differential expression at PSBs from stage 7 onwards, and the removal of *comm* on its own shows a modest contribution to interfacial contractility at PSBs (Fig. 2H,I), so this is a potential candidate for a role in late axis extension. Comm may act redundantly with Comm2, and together regulate Robo or another cell surface receptor. *Toll-6* and *Toll-8* are also expressed differentially at PSBs, at least some of the time (Fig. S3), but previous reports showed that these, along with *Toll-2*, do not contribute significantly to interfacial contractility at PSBs during GBE (Lavalou et al., 2021; Paré et al., 2019). During extended germband stages, *wingless* is required for maintaining interfacial contractility at PSBs (Monier et al., 2010; Scarpa et al., 2018; Urbano et al., 2018), and we confirm this property in this study, using a different quantification method (Fig. 3F,G). *Toll-2* was the only gene to respond to Wg signalling, out of 11 candidates we tested (*Best1*, *comm*, *comm2*, *dnt*, *drl*, *sli*, *Toll-2*, *Toll-6*, *Toll-7*, *Toll-8*, *trn*). However, we cannot detect a contribution of *Toll-2* on its own at PSBs, so the regulation of Toll-2 by Wg signalling does not readily explain the requirement for Wg at PSBs in maintaining interfacial contractility.

Like *Toll-2*, *Toll-6* and *Toll-8*, *tartan* is regulated transcriptionally by pair-rule genes at gastrulation. We confirm a previous report that *ftz* is required for expression of *trn* (Chang et al., 1993), but found that, although *trn* expression is lost at even-numbered PSBs in *ftz* mutants, it remains at odd-numbered PSBs. This might explain why odd-numbered PSBs in *ftz* mutants behave as in wild type in our boundary straightness analyses in live embryos. Thus, distinct transcriptional inputs regulate *trn* at even- and odd-numbered PSBs, but, despite this independent regulation, both boundaries behave similarly in wild-type embryos and both require *trn* in early axis extension for boundary straightness (Fig. S9A-C′).

Our *in silico* screen was based on the assumption that differential expression of receptors underlies interfacial contractility at PSBs. One limitation of our approach is that borders in mRNA expression detected by HCR do not necessarily equate with an asymmetry in protein localisation, because post-translational regulation could modulate receptor localisation. However, this approach was sufficient to identify Tartan. Also, comparison of protein and mRNA expression patterns for *tartan* and *comm* suggests that these are comparable, with the main difference being that the mRNA pattern is ahead temporally compared with protein. For example, at the beginning of GBE *tartan* mRNA expression retracts away from the odd-numbered PSBs (as does *ftz*), but the protein pattern is still abutting the PSBs (Fig. S4). Recent reports have identified additional cell surface molecules that interact with LRR receptors at boundaries and become asymmetrically localised (Lavalou et al., 2021; Paré et al., 2019). Those are the cell surface molecules Ten-M (a teneurin) and Cirl (an adhesion G protein-coupled receptor), which become localised at boundary cell-cell interfaces via their interactions with Tartan and Toll-8, respectively (Lavalou et al., 2021; Paré et al., 2019). Ten-M and Cirl have a uniform RNA expression in embryos and thus were eliminated as candidates in our *in silico* screen (Fig. S2). The planar polarisation of those heterophilic receptor complexes is thought to underlie the formation of contractile cell-cell interfaces, via pathways which are starting to be elucidated (Garcia De Las Bayonas et al., 2019; Tamada et al., 2021).

Our study shows that the measure of straightness is a sensitive and faithful read-out for increased actomyosin contractility along a boundary and provides an alternative to Myosin II quantifications. Our analysis in live embryos also suggests that mechanical boundary formation responds in real time and with high sensitivity to molecular asymmetries, because the recovery of PSB straightness in *tartan* mutant parallels the loss of expression of *tartan* along PSBs. It is likely that mechanosensitive feedbacks contribute to this responsiveness; indeed, Myosin II-enriched cell interfaces connected to each other enrich more Myosin II and are under greater tension than isolated interfaces, both during GBE (Fernandez-Gonzalez et al., 2009) and extended germband stages (Scarpa et al., 2018), suggesting the existence of a positive mechanosensitive feedback. Consistent with this notion, in both cases, decreasing tension at connected cell-cell interfaces using laser cuts also decreases Myosin II enrichment (Fernandez-Gonzalez et al., 2009; Scarpa et al., 2018). Thus, it is possible that mechanosensitive feedback increases actomyosin enrichment along PSBs, contributing to the real-time responsiveness of boundary formation. This might also contribute to the robustness of boundary formation (Martin et al., 2021).

## MATERIALS AND METHODS

### *In silico* screen

To identify *Drosophila* genes expressed in AP stripes in the early embryo, the BDGP library (https://www.fruitfly.org/) was filtered using the descriptors ‘Pair-Rule’ and/or ‘Segmentally Repeated’, the Fly-FISH library (http://fly-fish.ccbr.utoronto.ca/) was filtered using the descriptors ‘Pair-Rule’ and/or ‘Segment Polarity’, the Enhancer Library (http://enhancers.starklab.org/) was filtered using the descriptors ‘A-P Stripes’ and/or ‘Pair-Rule’, and the vISH library (https://shiny.mdc-berlin.de/DVEX/) was manually interrogated for the expression pattern of 441 genes predicted to encode transmembrane adhesion protein in *Drosophila* (Hynes and Zhao, 2000). Manual clustering analysis of the vISH library raw data was also performed to identify the top 200 genes expressed in the same cells as those expressing *even-skipped* or *fushi-tarazu* in early embryos (personal communication from N. Karaiskos and R. Zinzen, Max Delbrück Center for Molecular Medicine, Berlin).

To identify genes encoding proteins that localise to the cell surface, the UniProt data resource (http://www.uniprot.org) was filtered for the descriptors: ‘annotation:(type:transmem) AND organism:“Drosophila melanogaster (Fruit fly)” ’ and also ‘annotation:(type:signal) AND organism:“Drosophila melanogaster (Fruit fly)” ’*.*

To identify genes regulated by the pair-rule gene network, differentially expressed genes resulting from the knock down of *even skipped* and *runt* in early *Drosophila* embryos were obtained from a previous report (Paré et al., 2014). Further, the Berkeley *Drosophila* Transcription Network Project (BDTNP) database was queried to identify genes neighbouring *fushi-tarazu*, *sloppy paired 1*, *paired* and *runt* DNA-binding sites, and the DroID database (http://www.droidb.org/Index.jsp) was used to identify genes neighbouring *even skipped*, *hairy* and *odd skipped* DNA-binding sites.

A custom R script was used to wrangle the downloaded filtered datasets into a standardised dataframe format and identified genes that fulfilled candidate criteria. The initial list of candidate genes was then trimmed as follows. (1) Each candidate had their raw *in situ* hybridisation images, contained in each library, manually assessed and if a gene was found not to be expressed in AP stripes, the gene was excluded. (2) The ModEncode temporal expression data set (Roy et al., 2010) (annotated version kindly provided by Nick Brown, University of Cambridge, UK) was used to exclude genes with less than 5% of their total embryonic expression [0-24 h after egg-laying (AEL)] occurring between 0-6 h AEL. (3) Finally, a manual investigation of protein localisation and described role was undertaken using FlyBase (Gramates et al., 2022) and a search in the primary scientific literature, to eliminate genes that were unlikely to have a direct role at the cell surface, such as transcription factors.

### Whole-mount *in situ* HCR v3.0

Two- to five-hours-old *yw^67^* embryos were collected on apple juice agar plates at 25°C, fixed in 4% formaldehyde/heptane for 20 min, and stored at −20°C in methanol until required. *In situ* HCR v3.0 with split initiator probes were performed as described by Choi et al. (2018). The probe sets were designed by Molecular Instruments to target exons present within every gene isoform. Embryos for whole-mount *in situ* HCR were first post-fixed in 4% formaldehyde, then washed in PBT (PBS with 0.1% Tween-20), then 5× SSCT prior to hybridisation. Embryos were pre-hybridised in warm hybridisation buffer for 30 min at 37°C. Embryos were incubated in the probe hybridisation solution (0.8 pmol of each probe in 200 μl) at 37°C overnight. Following overnight incubation, excess probes were removed by washing in wash buffer at 37°C, then in 5× SSCT at room temperature (RT). The embryos were pre-amplified in buffer then final amplification solution was added (6 pmol of each snap-cooled fluorescently labelled hairpin added to 50-100 μl of amplification buffer). Embryos were incubated in the amplification solution overnight then washed in 5× SSCT. Hybridisation, wash and amplification buffers are as described by Choi et al. (2018), with the exception that the concentration of dextran sulphate was halved to allow the embryos to sink. If antibody immunostaining was to follow, embryos were washed in PBT before being blocked in PBT with 1% bovine serum albumin (PBS-TX-BSA). Embryos were mounted in VECTASHIELD (Vector Laboratories) before imaging.

### Immunostaining and antibodies

Embryos were fast-fixed at the interface between 37% formaldehyde and 100% heptane for 8 min then washed thoroughly in PBS-TX. The vitelline membrane was either removed chemically using methanol or manually with a tungsten needle. Embryos were blocked in PBS-TX-BSA for 30 min at RT. Embryos were incubated with primary antibodies in blocking solution overnight at 4°C. Excess antibody was removed by washing embryos thoroughly in PBS-TX. Embryos were incubated with secondary antibodies in blocking solution for 1 h at RT. Excess antibodies were removed by washing thoroughly in PBS-TX. Stained embryos were stored in VECTASHIELD (Vector Laboratories) until mounted.

Primary antibodies used were: mouse anti-phospho-Tyrosine (pTyr) (Cell Signaling Technology, 9411; 1:1000), rat anti-DE-Cad [Developmental Studies Hybridoma Bank (DSHB); 1:50], rabbit anti-Engrailed (Santa Cruz Biotechnology, D300; 1:100), mouse anti-Wingless (DSHB; 1:25), chick anti-β-gal (Abcam, ab9361; 1:500), rabbit anti-Tartan (1:2500) (Chang et al., 1993) (kind gift of Shigeo Hayashi, RIKEN Center, Kobe, Japan), rabbit anti-Comm (1:50) (Tear et al., 1996) (kind gift of Guy Tear, King's College London, UK).

Secondary antibodies (used at 1/250) conjugated to fluorescent dyes were obtained from Jackson ImmunoResearch Laboratories, Invitrogen and Life Technologies. Streptavidin with Alexa Fluor 405 conjugate was from Thermo Fisher Scientific.

### Confocal imaging of fixed embryos

Embryos were mounted individually under a coverslip supported by a tape bridge on either side. This flattened the embryos sufficiently so that all cells were roughly in the same *z*-plane. *In situ* HCR-stained embryos and immunostained embryos were imaged on an inverted SP8 Confocal Microscope (Leica Microsystems), with either a 20×0.75 NA air objective, 40×1.3 NA oil-immersion objective or 63×1.4 NA oil-immersion objective. Either a PMT or HyD detector was used alongside a 405/488/546/594/647 nm laser line. Image stacks of various *z* separations were captured using Leica Application Suite X Software.

### Embryo staging and mapping of expression patterns by HCR

Fixed embryos were staged initially based on their morphology under a light microscope, before mounting in VECTASHIELD. The staging was then refined when confocal imaging. Embryos with invaginated mesoderm but prior to the first mesectoderm cells dividing (identified by their dumbbell shapes) were classified as stage 7 or ‘early GBE’. Embryos past this stage and up to the time when the first neurectoderm cells divide were classified as stage 8 or ‘late GBE’. Embryos past this stage and up to the formation of tracheal pits were classified as ‘extended germband’ and included stage 9 and 10 embryos. This staging was used in the quantifications presented in Figs 2 and 3. Note that in our live embryos analyses, the first 30-40 min of GBE corresponds to stage 7/early GBE and the remainder, stage 8/late GBE (for example, see Fig. 5D).

To classify embryos labelled by HCR, similar staging was used, with earlier embryos (stages 5 and 6) also included in the analysis. Candidate genes patterns were mapped relative to parasegmental boundaries using various markers by HCR (*ftz*, *slp1*, *en*, *wg*) and also membrane immunostaining (pTyr or DE-Cad, both labelling adherens junctions). This work is summarised in diagrams showing representative odd- and even-numbered parasegments separated by odd-numbered or even-numbered PSBs (for example, see Fig. S3). For simplicity, each parasegment was divided into four regions for each of the developmental stages analysed, but it is important to note that cell numbers along AP slowly increase through polarised cell intercalation and then cell division. At the start of GBE, the four regions correspond approximately to the number of cells per parasegment, which we measured as 3.7 cells on average (Tetley et al., 2016). Germband cells undergo one round of cell intercalation, bringing the parasegment width to 7.2 cells in average (Tetley et al., 2016). At the end of GBE, germband cells start dividing and the number of cells per parasegment increases further.

### Fly genetics

We used *yw^67^* as control. Null mutant alleles were used for the following genes: *fushi-tarazu* (*ftz^11^*; embryonic lethal, pair-rule cuticle phenotype, see Fig. S9E); *wingless* (*wg^CX4^*; embryonic lethal, segment polarity cuticle phenotype); *tartan* [*trn^28.4^* (Chang et al., 1993) for quantifications; *trn^S064117^* for complementation tests (embryonic lethal)]; *Toll-2* [*toll-2^Delta7-35^* (Eldon et al., 1994) for quantifications; *toll-2^K02701^* (Yagi et al., 2010) and *toll-2^pTV^* (Li et al., 2020) for complementation tests (embryonic semi-lethal, escapers have abnormal climbing behaviour; Li et al., 2020)]; *commissureless* [*comm^Delta e39^*; https://flybase.org/reports/FBal0097023 (gift from Jimena Berni, University of Cambridge, UK) for quantifications. Note that *comm^Delta e39^* is the same allele as *comm^E39^* (https://flybase.org/reports/FBal0141222) (G. Tear, personal communication), which is a deletion of the *comm* transcription unit (Georgiou and Tear, 2002). We used *comm^A990^* (gift from Guy Tear) and *Df(3L)BK10* for complementation tests (embryonic lethal).

Transgenes were: *Gap43mCherry* (Martin et al., 2010) to label cell membranes, *sqh^EGFP.29B^* (called *sqhGFP^KI^* here) (Proag et al., 2019) to label Myosin II, *EnVT15159-peve-MS2-lacZ* (this work), *nos-MCP-eGFP* on II (Garcia et al., 2013), *armGal4* (Sanson et al., 1996) and *UASwg* (Lawrence et al., 1996).

Balancer chromosomes used for homozygous embryo selection were: *CyO, evelacZ* or *TM6C, twilacZ* (for fixed embryos) and *TM3, twiGal4,UASGFP* (TTG, for live embryos).

### Quantification of Myosin II intensities and boundary straightness at PSBs

Quantifications were performed in fixed embryos using the following fly strains. Fig. 2E,F: *sqhGFP^KI^; trn^28.4^/TM6C, twilacZ*; Fig. 2H,I: *sqhGFP^KI^; comm^Delta e39^/TM6C, twilacZ*; Fig. 3D,E: *sqhGFP^KI^; toll-2^Delta7-35^/CyO, evelacZ*; Fig. 3F,G: *sqhGFP^KI^; wg^CX4^, enlacZ/CyO*. Homozygous embryos were identified based on absence of immunostaining against β-galactosidase except for *wg* null mutant embryos, for which loss of Engrailed immunostaining was used. The remainder of the embryos in the progeny were used as controls (wild type).

Using the plug-in Simple Neurite Tracer in Fiji (https://imagej.net/plugins/snt/), lines 2 pixels in width were traced along the PSB and control +1 cell-cell interfaces, based on Engrailed or Wingless (or *enlacZ* for *wg* null mutants) and pTyr or DE-Cad immunostainings (see traces in Fig. 2B). Native fluorescence from *sqhGFP^KI^* was then quantified in the corresponding traces. Normalisation was performed by removing background pixels using a threshold corresponding to 20% of total pixels. Ratios of PSB interface signal (background/+1 interface signal− background) were expressed on a log10 scale. Traces corresponding to PSBs were also used to calculate an index of straightness by dividing the length of the shortest path between the extremities of the trace and the length of the trace. Embryos of stages 7 to 10 were analysed. PSBs with boundary cells dividing were excluded from the analysis (see blue traces in Fig. 2B,C). As a consequence of the frequency of cell divisions, fewer PSBs of stage 9 were included in the analysis compared with stage 10 for extended germband stages. Graphs were made in R using ggplot2 library geom-boxplots.

### dsRNA generation

Design of dsRNA was based upon the Heidelberg 2 (BKN) library (Horn and Boutros, 2013). First, to generate transcription templates for production of dsRNA, a PCR was undertaken on *yw^67^* fly gDNA using a Q5 polymerase master mix (NEB) and the following primer pairs (preceded by the T7 promoter sequence: 5′-TAATACGACTCACTATAGGG-3′): *fushi-tarazu* forward 5′-CCGCCCACCTACTACGATAA-3′, reverse 5′-CAGCTGACGAGGATTTCTCC-3′; 577 bp length.

ssRNA was transcribed directly from the PCR amplicon product in a reverse transcription reaction using a HiScribe T7 polymerase (NEB). The DNA template was then removed through treatment with DNase I. ssRNA was annealed to form dsRNA through addition of 0.5 M EDTA, 10% SDS and 3 M NaCl, boiling the mixture then cooling to RT naturally. Annealed dsRNA was purified through a standard phenol:chloroform:IAA 25:24:1 extraction and precipitated from solution by adding of ethanol and ammonium acetate. The isolated dsRNA pellet was washed multiple times in 70% ethanol, air dried, and resuspended in injection buffer (0.1 mM sodium phosphate buffer, 5 mM KCl). The dsRNA was injected into pre-cellularised embryos at a concentration of 1.7 μg/μl, as measured by nanodrop.

### Embryo injections

Adult flies were kept at 25°C in a cage with an apple juice agar plate. Embryos were collected from the plate following a 30-min laying period and were dechorionated. Approximately 20 embryos (for dsRNAi experiments) and 100 embryos (for transgenic injections) were aligned on a block of agar and transferred to a coverslip using a thin layer of heptane glue. If the injected embryos were for live imaging, embryos would be aligned with their ventral side facing the glue and coverslip. Embryos were desiccated in a jar of silica beads for 10-12 min before being covered with a thin layer of VOLTALEF (PCTFE H10S, Arkema). A brightfield microscope (Olympus CK40), microinjection apparatus (Leitz), and a pulled glass needle were used to inject the embryos through their posterior end. For RNA-interference experiments, the expulsion of dsRNA was aimed at the centre of the embryo. For the generation of transgenic flies, plasmid solution was injected at the posterior end of the embryo (where the future pole cells form). Unfertilised, damaged, or old embryos were destroyed with forceps. Slides of injected embryos were placed in a 50 mm Petri dish at 18°C until the correct stage of development.

### Cuticle preparation

Dechorionated embryos were transferred to a 50:50 mix of Hoyer's medium and lactic acid and mounted under a 22×32 mm coverslip. A permanent marker was used to draw black dots onto the surface of the coverslip to help locate embryos for microscopy. The slide was baked overnight at 62°C with a weight on top of the cuticle to prevent air bubbles forming. Cuticle preps were imaged using darkfield or phase-contrast microscopy.

### EnVT15159-MS2 generation

The Stark Lab fly enhancer library was used to identify a small region of the *engrailed* enhancer that accurately recapitulates expression at germband extended stages of embryogenesis. Tile ID VT15159 contained a 2099 bp region of DNA that neighbours the *engrailed* gene and drives *lacZ* in an *engrailed* pattern. The 2099 bp region (EnVT15159) was PCR amplified from purified *yw^67^* gDNA using Q5 DNA polymerase MasterMix (NEB) and the following primers: EnVT15159 forward 5′-GGG[AAGCTT]GGCGTTTGTGGGGATGTTTCAAGTTG-3′, reverse 5′-GGG[ACCGGT]TCTTAGCCAGGCTTGTTAGCCGC-3′. Square brackets indicate HindIII (forward primer) and AgeI (reverse primer) restriction enzyme cut sites. Primers were designed so the restriction cut site was preceded by three guanine bases.

To confirm successful amplification of the EnVT15159 region, 2 μl of the PCR product was run on a 1% agarose gel. The PCR product was cleaned using the QIAquick PCR clean-up kit (QIAGEN). The EnVT15159 PCR product was then digested using HindIII and AgeI high fidelity restriction enzymes (NEB) in Cutsmart buffer (NEB).

The digested EnVT15159 region was cloned into *pattB-w+-pEve-24xMS2-lacZ* plasmid (kind gift of Julia Falo-Sanjuan and Sarah Bray, University of Cambridge, UK) using the HindIII and AgeI sites. The plasmid was transformed into DH5-alpha library-efficiency competent cells (Invitrogen) through a standard heat-shock protocol. Transformed colonies (displaying ampicillin resistance) were picked, grown into 50 ml of culture and isolated via a MaxiPrep kit (QIAGEN). Plasmid fingerprinting was undertaken using BbsI and EcoRV restriction enzymes (NEB) to confirm the EnVT15159 product had been inserted into the plasmid in the correct orientation. We injected the final plasmid construct *pattB-w+-EnVT15159-pEve-24xMS2-lacZ* into *yw, M(eGFP, vas-int, dmRFP)ZH-2A;; M(attP)ZH-86Fb* flies (sourced from Genetics Department Fly Facility, University of Cambridge, UK). The construct was inserted by phiC31-mediated integration into the *attP-86Fb* site on the third chromosome (86F8). F1 Transgenic flies were identified through the presence of w+. Crosses were undertaken to generate *yw;;EnVT15159-MS2* flies that were homozygous viable and established as a stable stock.

### Live imaging

Dechorionated embryos were mounted using an adapted hanging drop methodology (Reed et al., 2009). Briefly, a 22×64 mm coverslip (#1) was attached to a rectangular metal microscope slide frame (Leica) using Magic Tape (Scotch). Live embryos freely suspended in VOLTALEF (PCTFE H10S, Arkema) were positioned with their ventral side towards the coverslip. The frame and coverslip were quickly inverted. The ventral side of the embryo remained in contact with the coverslip. Embryos were imaged under a 40× oil objective lens (NA 1.3) on a Nikon Eclipse E1000 microscope with a Yokogawa CSU10 spinning disc head and a Hamamatsu EM-CCD camera. Embryos were illuminated using a Spectral Applied Research LMM2 laser module (491 nm and 561 nm excitation). Images were captured using Volocity Acquisition Software (Perkin Elmer); 32 *z*-slices with a 1 μm separation were obtained at each time point. Embryos were imaged every 30 s from late stage 5 for 100 min. Movies were recorded at 20.5±1°C, measured with a high-resolution thermometer (Checktemp1, Hanna Instruments). To check that embryos survived the imaging process to the end of embryogenesis, embryos were allowed to develop on the imaging insert to hatching in a humidified box. For mutants that are embryonic lethal, the cuticle of embryos was prepared using standard methods to check their phenotype. Occasional movies acquired for embryos that did not hatch or did not make a cuticle at the end of embryogenesis were discarded.

### Genetic tools for boundary-straightness analysis in live embryos

We used progeny embryos from females *Gap43-mCherry/CyO; Nos-MCP-GFP/TM6B* or *Gap43-mCherry/CyO; Nos-MCP-GFP, trn^28.4^/TTG* crossed with males *yw^67^;;EnVT15159-MS2* or *yw^67^;;EnVT15159-MS2, trn^28.4^/TTG*. Note that there is sufficient maternal contribution of Gap43-mCherry to have a strong membrane signal in progeny embryos.

In Fig. 5, for the comparison between wild-type and *trn* mutant embryos, *trn* homozygous embryos were identified by the lack of *twiGal4,UASGFP* fluorescence by mid-embryogenesis and wild-type embryos were the *twiGal4,UASGFP*-positive embryos.

In Fig. 6, embryos were *Gap43-mCherry/+; Nos-MCP-GFP/EnVT15159-MS2* injected with either buffer (BI) or dsRNA against ftz (*ftz* KD).

### Cell-tracking analysis

Cell tracking based on the Gap43-mCherry membrane signal, spatiotemporal movie synchronisation, domain strain rate calculations, cell selection criteria and contoured heat map generation were performed as described by Tetley et al. (2016).

### Defining PSB interfaces and cell types

Tissue domains were defined in individual tracked movies by examining the position of EnVT15159-MS2/MCP-GFP transcriptional dots. To assign dots to cells, cell tracking was undertaken using custom software (Blanchard et al., 2009) on movies of embryos containing EnVT15159-MS2, MCP-GFP and Gap43-mCherry. First, the MS2-MCP signal was processed, and a pixel intensity threshold applied to identify dots in an automated manner. Next, the Gap43-mCherry signal was processed and a blanket correction applied to uncurve the 3D surface of the embryo. A few slices of the Gap43-mCherry signal, just under the apical surface of cells, were maximum intensity projected so cell outlines were clear and individual cells could be segmented. Segmented cells were tracked back and forth through time and each cell was marked with a unique identity. Fluorescent transcription dots (resulting from MS2-MCP binding) were also tracked back and forth through time and each dot was assigned to a corresponding cell. Based upon the assignment of dots, *engrailed*-expressing cells were identified and cells could then be classified into parasegments meaning PSB interfaces could also be identified. Because cells were tracked over time, these classifications of parasegment identity could be automatically tracked through time to define the same groups of cells at all earlier and later time points.

### Quantification of interface co-alignment

Interface orientations, relative to the embryonic axes, were calculated for PSB, −1 and +1 in all movies. All distributions of interface orientations (from 0, parallel to the AP embryonic axis, to 180°) were reflected around 90°, producing distributions from 0° (AP aligned) to 90° (DV aligned). As a measure of co-alignment, the proportion of interfaces oriented between 60 and 90° relative to the AP axis was plotted over time, from −10 to 50 min. Graphs were produced in R. For the LOESS curves (https://rdrr.io/r/stats/loess.html), the following library in R was used: https://ggplot2.tidyverse.org/reference/geom_smooth.html#details. Cumulative frequencies were calculated for each reflected distribution of interface orientations. Two-sample Kolmogorov–Smirnov tests on the cumulative frequency distributions of interface orientation were used to compare treatments/genotypes.

## Supplementary Material

10.1242/develop.200292_sup1 Supplementary information
