# Supplementary material for: Different temporal requirements for tartan and wingless in the formation of contractile interfaces at compartmental boundaries
Source: Development. 2022 Oct 31;149(21):dev200292. doi: 10.1242/dev.200292 (PMC9687003; doi:10.1242/dev.200292)
Supplement: Supplementary information [file develop-149-200292-s1.pdf]

Figure S1.

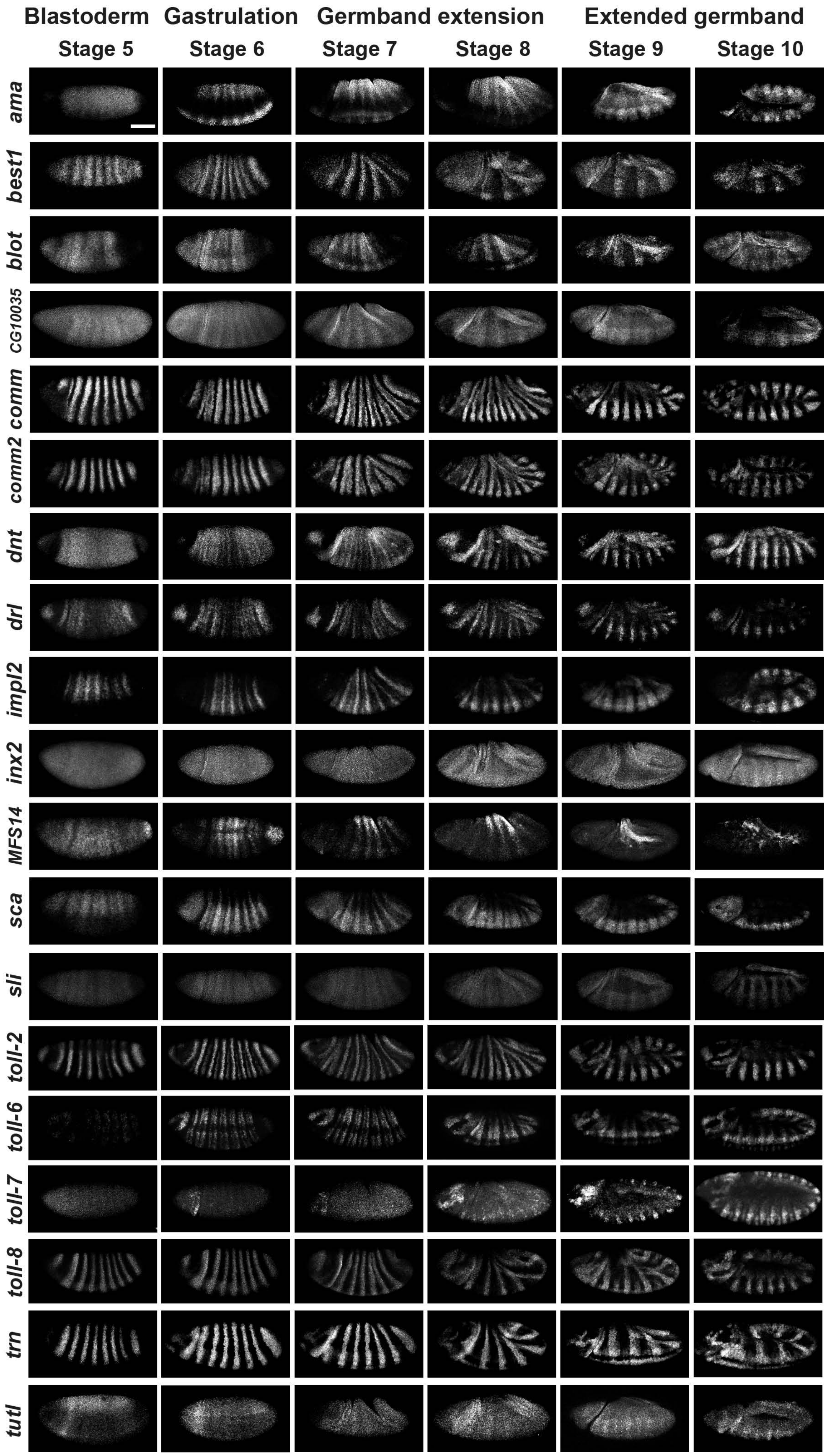

Development • Supplementary information

Fig. S1. *In situ* HCR for candidate genes with antero-posterior striped expression. HCR was performed for each gene in *yw*^{67} *Drosophila* embryos between stages 5 to 10 of embryogenesis. The 19 genes included in this figure were classified as having a striped expression pattern at some point during stages 5-10. For some genes, such as *tutl*, subtle stripes are only clearly visible at a subset of the stages surveyed (stages 9-10), whereas other genes, such as *trn*, have clear expression stripes visible at all stages. Scale bar = 100μm.

Development • Supplementary information

Figure S2.

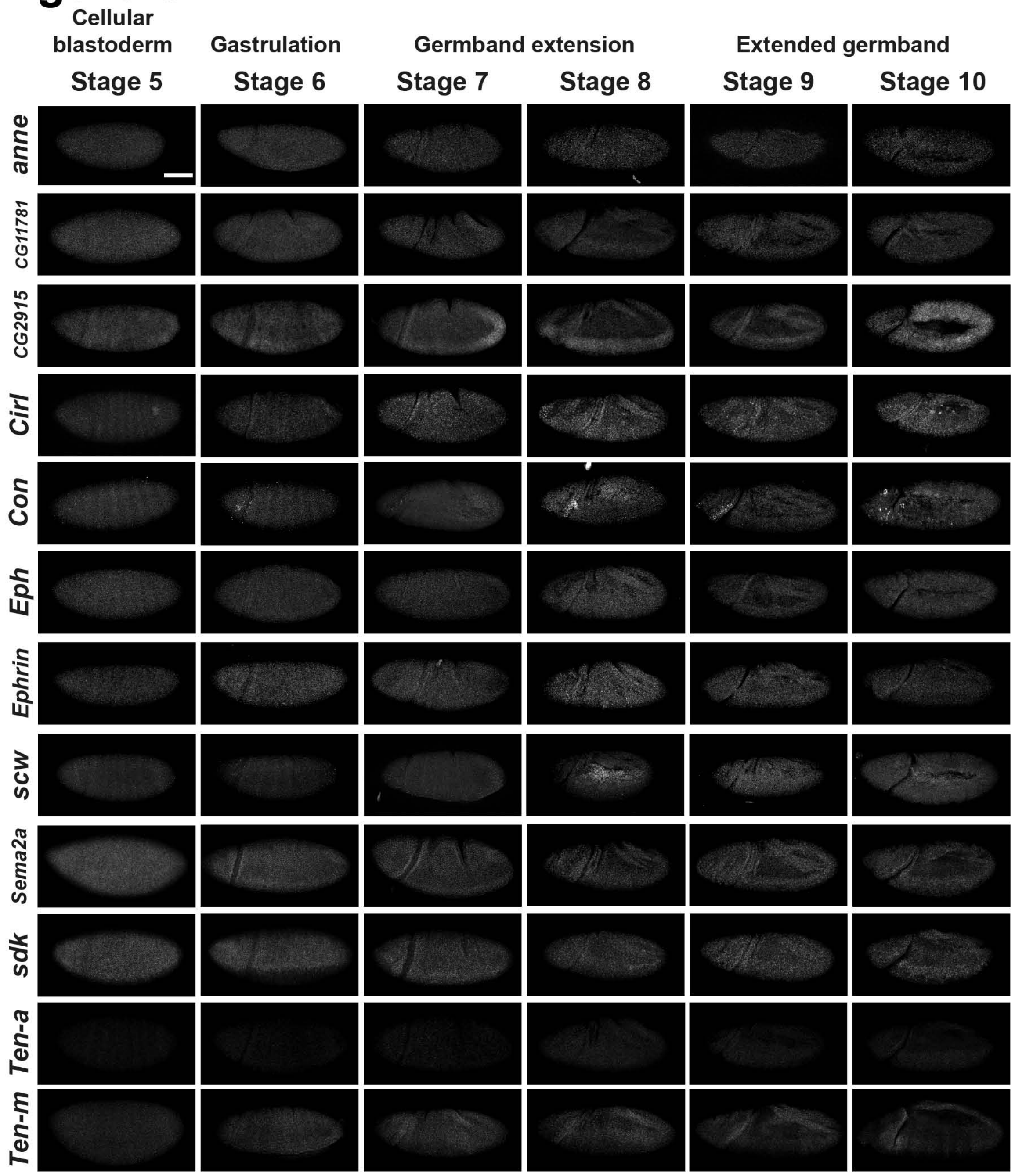
Cellular blastoderm
Gastrulation
Germband extension
Extended germband
Stage 5
Stage 6
Stage 7
Stage 8
Stage 9
Stage 10
anne
CG11781
CG2915
Cirl
Con
Eph
Ephrin
scw
Sema2a
sdk
Ten-a
Ten-m

Fig. S2. *In situ* HCR for candidate genes without AP striped expression pattern. HCR was performed for each gene in yw^{67} *Drosophila* embryos between stages 5 to 10 of embryogenesis and the 12 genes included here were found not to have any AP striped expression. Scale bar = 100µm.

Development • Supplementary information

Figure S3.

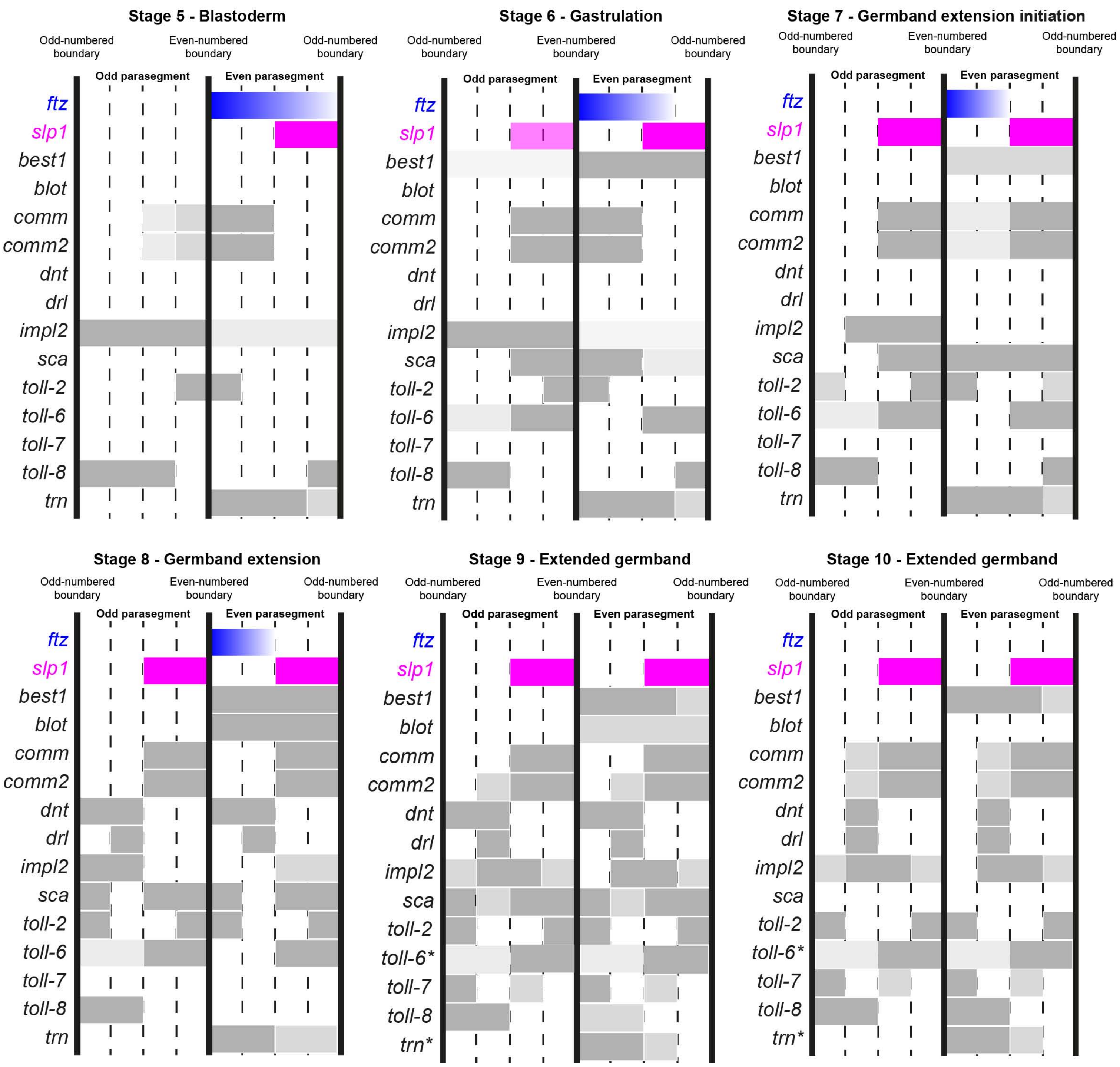

Development • Supplementary information

Fig. S3. Map of expression patterns of candidate genes relative to the PSBs. The maps are drawn based on gene expression patterns revealed by HCR for each candidate genes in combination with the parasegmental boundary markers *ftz* and *slp1*. HCR was performed in *yw*^{67} *Drosophila* embryos between stages 5 to 10 of embryogenesis. Representative expression for odd- and even-numbered parasegments are shown. Rather than trying to represent cell numbers, we have divided each parasegment into 4 regions, for simplicity of comparison across the different developmental stages (see Methods). Out of the 19 candidates with AP striped expression shown in Sup. Fig. 1, the 13 genes included here showed expression patterns that were clear enough to enable mapping relative to the PSBs.

*Note that for both *tartan* and *toll-6*, expression becomes discontinuous along DV.

Development • Supplementary information

Figure S4.

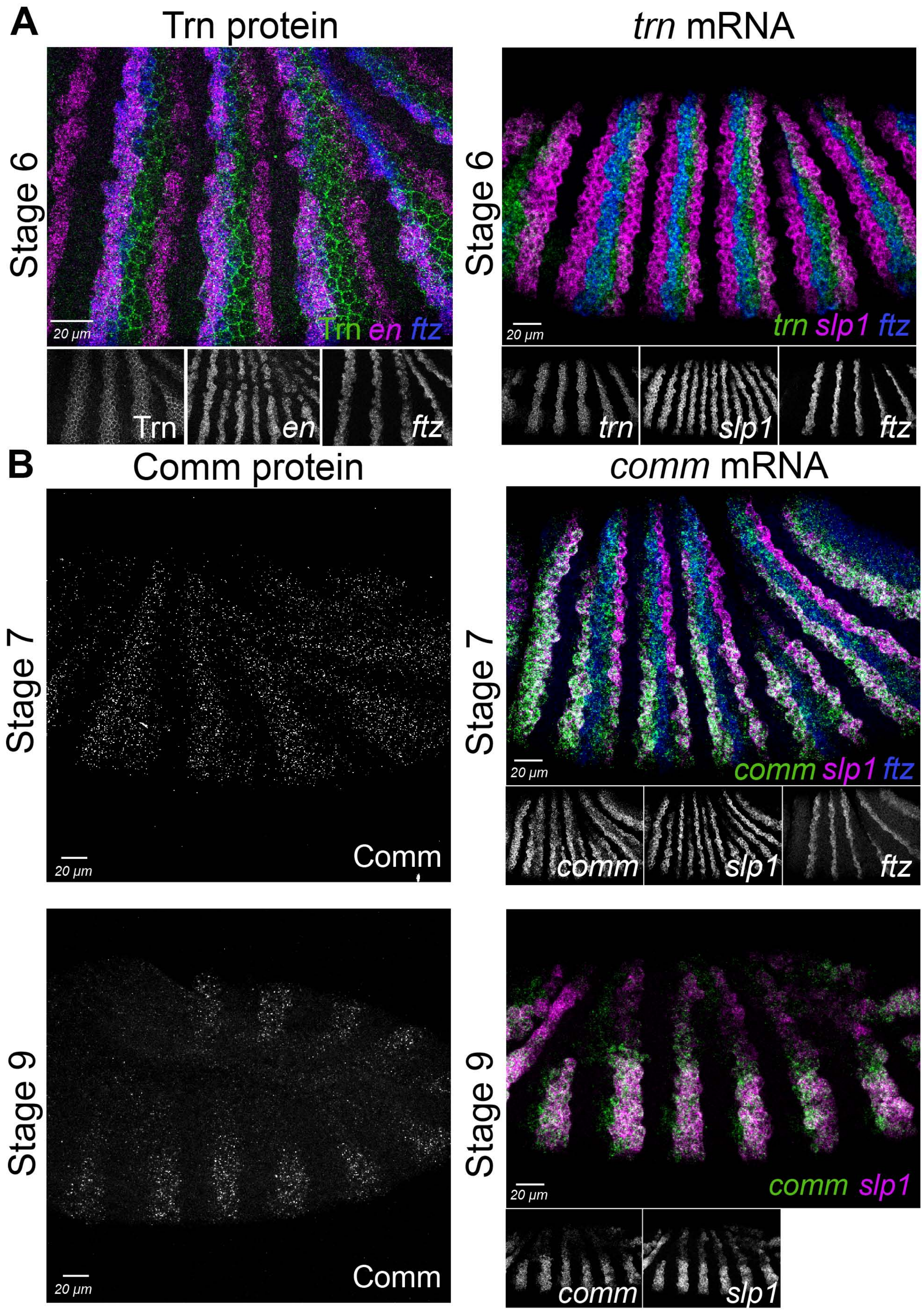
A
Trn protein
trn mRNA
Stage 6
Stage 6
20 µm
Trn en ftz
20 µm
trn slp1 ftz
Trn
en
ftz
trn
slp1
ftz
B
Comm protein
comm mRNA
Stage 7
Stage 7
20 µm
Comm
20 µm
comm slp1 ftz
comm
slp1
ftz
Stage 9
Stage 9
20 µm
Comm
20 µm
comm slp1
comm
slp1

Fig. S4. Comparison of mRNA and protein patterns for candidate genes *tartan* and *comm*. A) Left panel shows an immunostaining against the Tartan protein in combination with HCR for *en* and *ftz*. Right panel show HCR for *tartan,* in combination with *slp1* and *ftz*. Both images show a stage 6 yw^{67} embryo. For the immunostain, maximum intensity projection are shown for the full z-depth of the *engrailed* and *fushi-tarazu* channels and for 2µm of Tartan signal, just below the apical surface of cells. Tartan protein localises to the membrane of cells located within even-numbered parasegments and is absent from cells within odd-numbered parasegments at stages 6-8 of embryogenesis. B) Left panel show immunostainings against Comm protein and right panel HCR for *comm*, in combination with *slp1* and *ftz* markers. Images of stage 7 and 9 yw^{67} embryos show that *comm* patterns for both mRNA and protein undergo a doubling of periodicity (see also Sup. Fig. 3).

FigureS5.

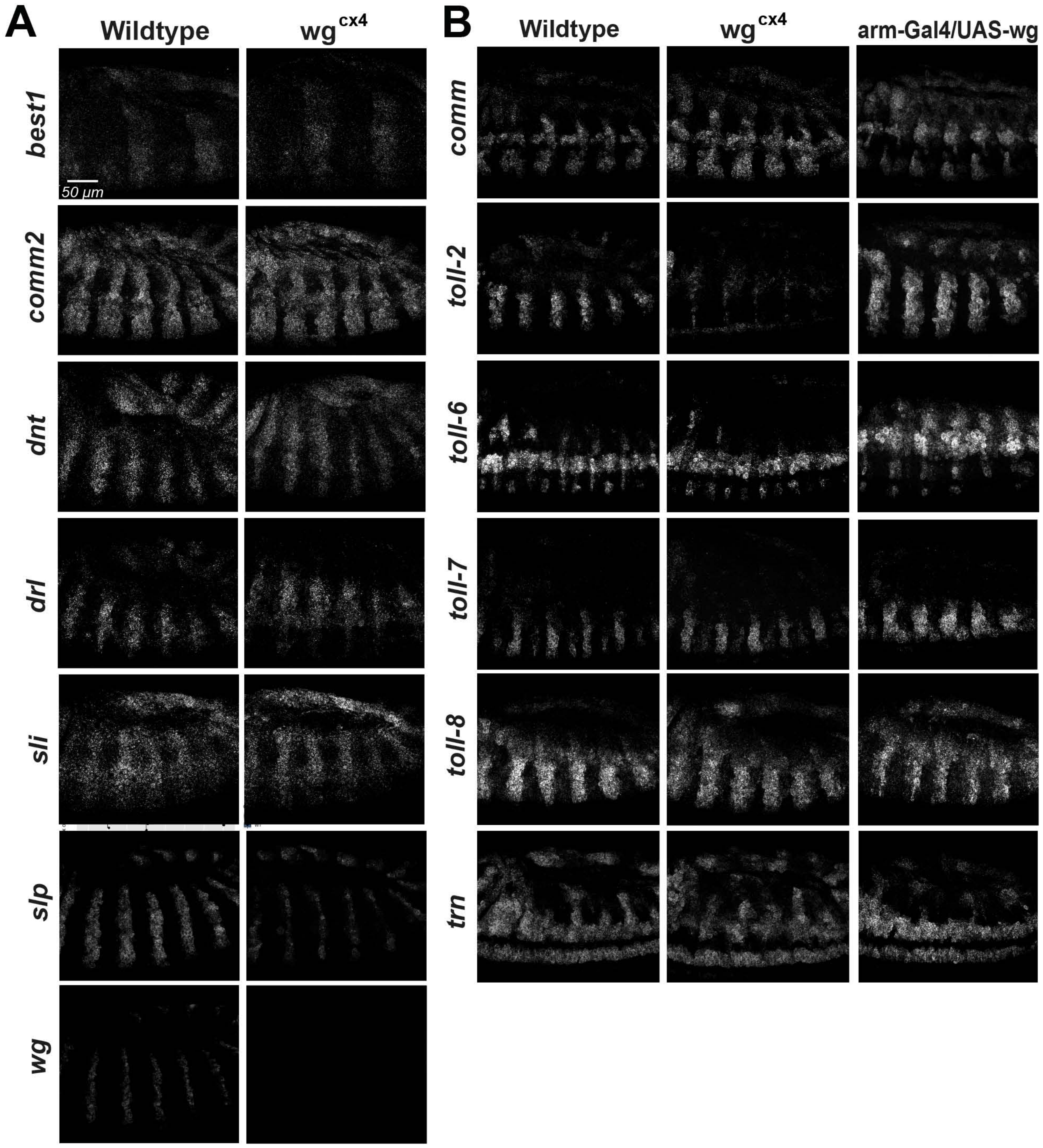
A
Wildtype
wg cx4
best1
50 µm
comm2
dnt
drl
sli
slp
wg
B
Wildtype
wg cx4
arm-Gal4/UAS-wg
comm
toll-2
toll-6
toll-7
toll-8
trn

Fig. S5. *In situ* HCR to identify candidate genes regulated by Wingless signalling. A) HCR for a subset of candidate genes (*best1*, *comm2*, *dnt*, *drl*, *sli*) in WT and wg^{cx4} mutant embryos, alongside *wg* and *slp1* controls**.** B) HCR for an additional subset of candidate genes (*comm*, *toll-2*, *toll-6*, *toll-7*, *toll-8*, *trn*) in WT, wg^{cx4} mutant embryos and in *armGal4/UASwg* embryos expressing Wg ubiquitously. Out of all the genes tested, only *toll-2* shows robust change in HCR, with loss of signal in wg^{cx4} mutant and increase in *armGal4/UASwg* embryos.

Figure S6.

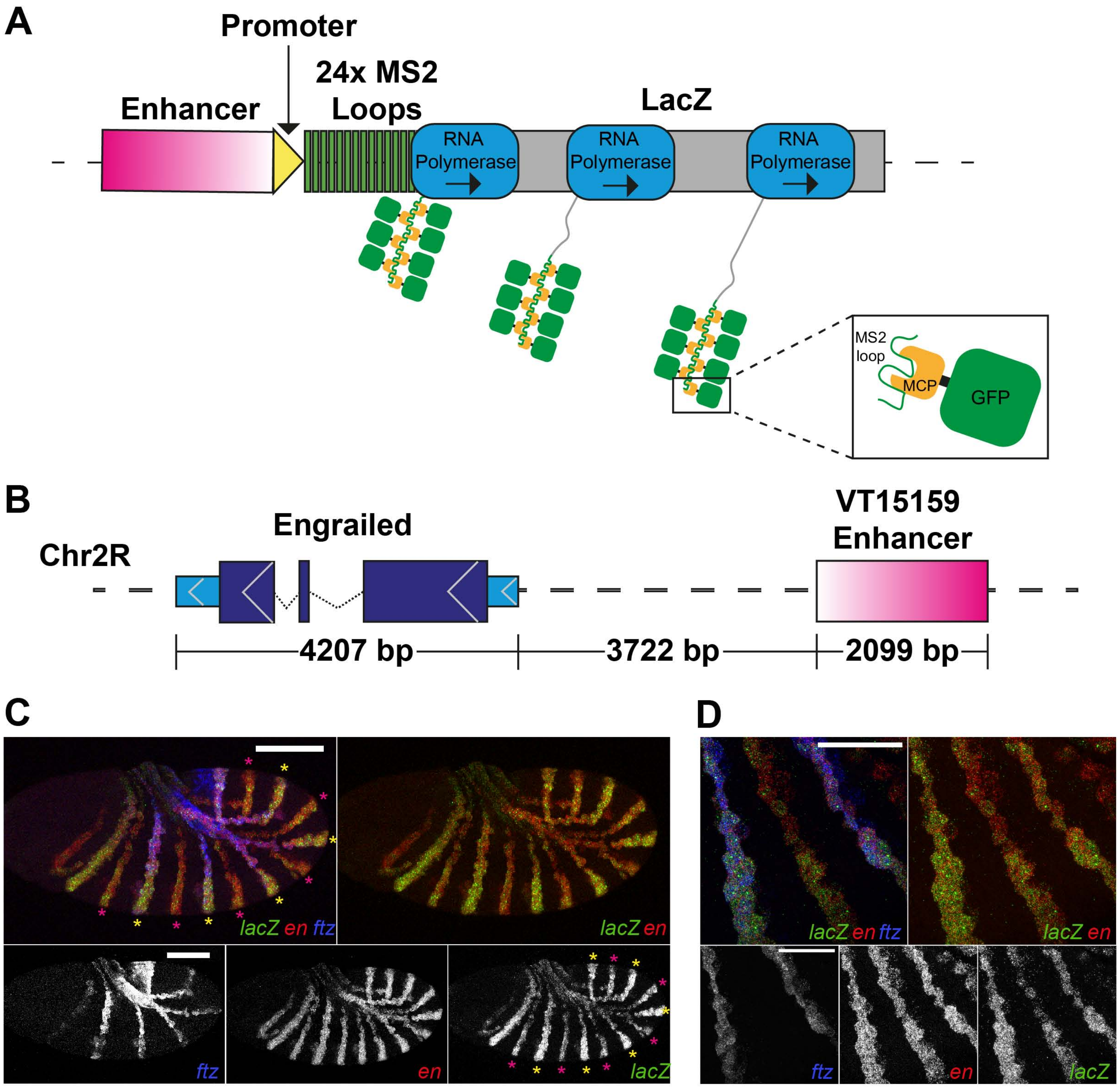
A
Promoter
24x MS2
Loops
Enhancer
LacZ
RNA
Polymerase
RNA
Polymerase
RNA
Polymerase
MS2
loop
MCP
GFP
B
Engrailed
VT15159
Enhancer
Chr2R
4207 bp
3722 bp
2099 bp
C
lacZ en ftz
lacZ en
ftz
en
lacZ
D
lacZ en ftz
lacZ en
ftz
en
lacZ

Fig. S6. MS2-MCP system to image nascent mRNA transcripts from an *engrailed* enhancer in live embryos. A) Diagram showing the MS2-MCP system. The gene of interest's enhancer region is cloned upstream of a strong promoter that initiates transcription. RNA polymerase transcribes the 24 MS2 stem loops followed by *lacZ*. The resulting mRNAs contains 24 copies of the MS2 loops that are bound by the MCP protein fused to GFP (box). The concentration of multiple MCP-GFP/ MS2 loop complexes causes a spatially localised fluorescence that is detected at the locus as a fluorescent dot. Multiple transcripts are produced from a single locus simultaneously increasing the brightness of the fluorescent dot. *lacZ* transcription is accessory to the core system but prolongs the time the nascent mRNA stays within vicinity of the locus, making the fluorescent dot brighter. B) Gene diagram showing the position of the VT15159 enhancer region, which is located 3722bp upstream of the *engrailed* gene (adapted from the NCBI genome data viewer). C) HCR of *ftz* (blue), *engrailed* (red) and *lacZ* (green) in embryos transgenic for EnVT15159-MS2. *lacZ* expression from EnVT15159-MS2 coincides with endogenous *engrailed* expression demonstrating that EnVT15159 recapitulates endogenous *engrailed* expression during early embryogenesis. Note that brighter alternate *lacZ* stripes overlap with *ftz* expression, indicating differential patterning by the VT15159 enhancer between odd- and even-numbered parasegments, with the brighter stripes abutting even-numbered PSBs. (20x magnification. Scale bar = 100μm) D) Close-up to show the coincidence between endogenous engrailed expression and *lacZ* expression from EnVT15159. The brighter *lacZ* stripes overlap with *ftz* expression and abut even-numbered PSBs. (63x magnification and 2x zoom. Scale bar = 50μm)

Development • Supplementary information

Figure S7.

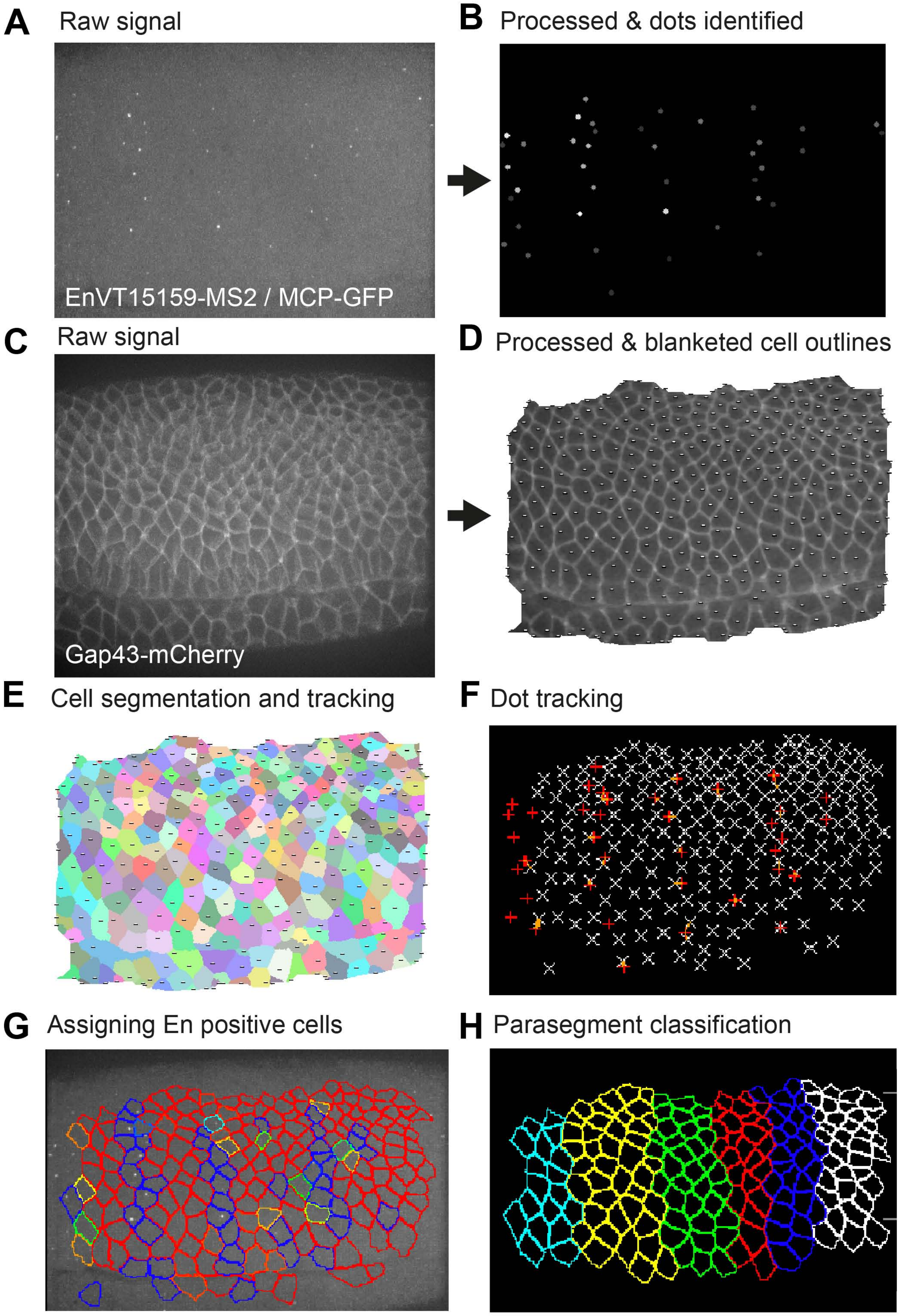

Development • Supplementary information

Fig. S7. Image processing and cell tracking pipeline to identify parasegmental boundaries in live embryos. A,B) Raw EnVT15159-MS2-MCP-GFP signal is processed in the custom software oTracks. A median background subtraction of 10 is applied. Fluorescent dots are identified in an automated manner by applying a pixel intensity threshold. C,D) Raw Gap43-mCherry signal is processed within oTracks. Median smoothing of 1 is applied and the corners of the image are brightened to correct for microscope artefacts. A blanketing operation is applied to correct for the 3D curvature of the embryonic volume. A 2μm z-plane is projected from the embryonic volume onto a flat 2D surface for image segmentation. E) oTracks segments each cell based on the Gap43-mCherry signal using a watershed algorithm. Each cell is tracked back and forth through frames and connected in time. F) Identified dots are tracked back and forth through time in oTracks. Dots are assigned a probability of belonging to each cell by determining the proximity of each dot to each cell during the lifetime of each dot. G) The probability of a cell being assigned to a dot can be displayed as a color (red = low probability, blue = high probability). Cells with a high probability of containing a dot form AP stripes throughout the embryo similar to the *engrailed* expression pattern. H) Based upon the color coding in G, parasegment identities can be assigned to each cell and the location of PSBs can be identified.

Development • Supplementary information

Figure S8.

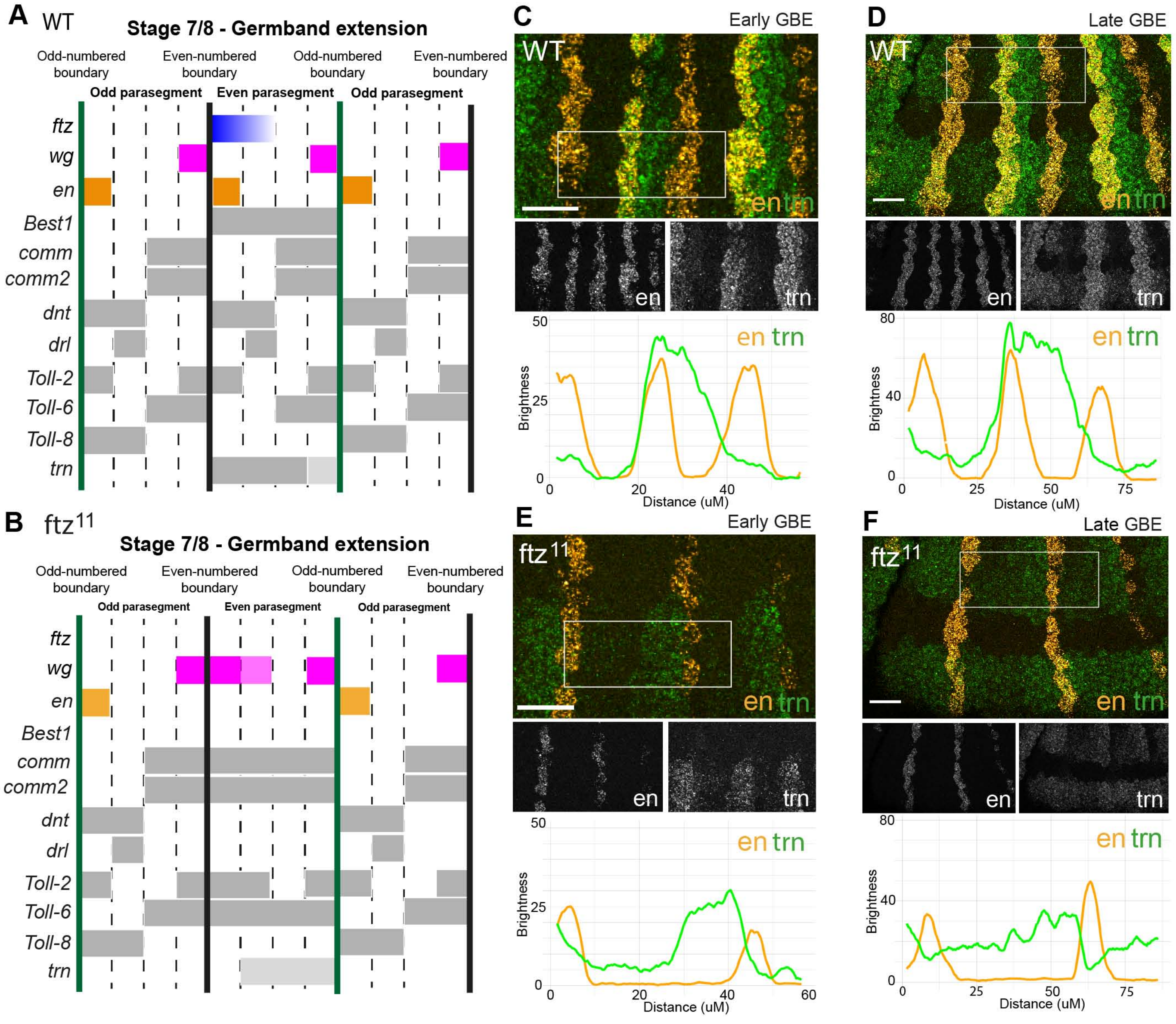

Development • Supplementary information

Fig. S8. Analysis of *tartan* expression at odd-numbered PSBs in *ftz* mutants. A, B) Diagrams summarising expression patterns by HCR of *best1, comm, comm2, dnt, drl, toll-2*, *toll-6*, *toll-8* and *trn*, in relation to PSB markers *ftz*, *wg* and *en*, in wildtype and *ftz* mutant embryos. *ftz* mutant embryos are homozygous for the *ftz*^{11} null allele while wildtype embryos are the heterozygous embryos in the same progeny. *ftz* homozygous mutant embryos were identified based on the changes in *en* expression pattern. C-F) Quantification of *tartan* HCR at even- and odd-numbered PSBs in WT and *ftz* null mutants. The rectangles show the regions of interest where *tartan* and *en* HCR signal has been quantified. In WT, *engrailed* stripes mark both odd- and even-numbered PSBs, whereas in *ftz* mutants, only the *engrailed* stripes at odd-numbered parasegments remain. "Early GBE" corresponds to stage 7 embryos; "late GBE", to stage 8. Scale bars =20 μm

Figure S9.

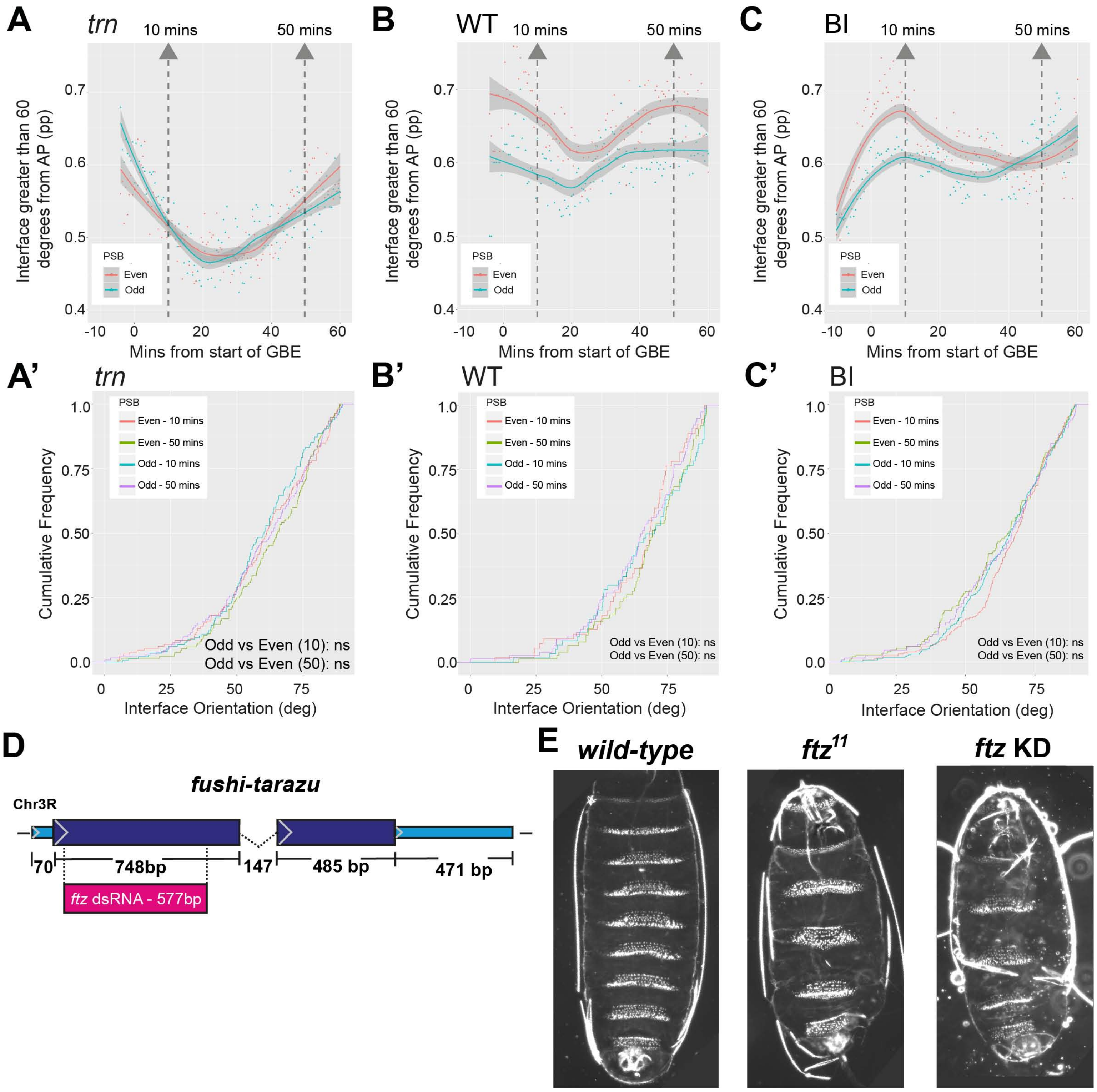

Development • Supplementary information

Fig. S9. Boundary straightness comparisons between even and odd-numbered PSBs and *ftz* dsRNA knockdowns A-C) Plots showing the proportion of odd- and even-numbered PSB boundary interfaces that are greater than 60 degrees from AP in 3 *tartan* mutant (A,A'), 3 wildtype (B,B') and 4 buffer injected embryos (C, C'). Odd and even-numbered PSBs are distinguished by the differential expression of EnVT15159-MS2 transcriptional dots. A loess curve (span 0.75) has been fitted to the data. Statistical comparison is a Kolmogorov-Smirnov non-parametric test undertaken on the cumulative frequencies of interface angles at 10 and 50 minutes (shown in A'-C'). D) *ftz* gene model (adapted from NCBI genome viewer) showing the dsRNA target sequence in exon 1 of *ftz* mRNA. E) Dark field microscopy images of cuticle preps from wildtype, ftz^{11} null and *ftz* dsRNA knockdown embryos. The *ftz* KD phenocopies the *ftz* null mutant, displaying the expected pair-rule phenotype.

Development • Supplementary information
