# Supplementary material for: Different temporal requirements for tartan and wingless in the formation of contractile interfaces at compartmental boundaries
Source: Development. 2022 Oct 31;149(21):dev200292. doi: 10.1242/dev.200292 (PMC9687003; doi:10.1242/dev.200292)
Supplement: Supplementary file 1 [file develop-149-200292_review_history.pdf]

Different temporal requirements for *tartan* and *wingless* in the formation of contractile interfaces at compartmental boundaries

Thomas E. Sharrock, Jenny Evans, Guy B. Blanchard and Bénédicte Sanson
DOI: 10.1242/dev.200292

Editor: Thomas Lecuit

Review timeline

Original submission:	21 October 2021
Editorial decision:	17 December 2021
First revision received:	7 July 2022
Editorial decision:	23 August 2022
Second revision received:	1 September 2022
Accepted:	12 September 2022

Original submission

First decision letter

MS ID#: DEVELOP/2021/200292

MS TITLE: Different temporal requirements for the LRR transmembrane receptors Tartan and Toll-2 in the formation of contractile interfaces at compartmental boundaries

AUTHORS: Thomas E Sharrock, Guy B Blanchard, Jenny Evans, and Benedicte Sanson

I very sincerely apologize for the long delay before being able to come back to you. I have now received all the referees reports on the above manuscript, and have reached a decision. The referees' comments are appended below, or you can access them online: please go to BenchPressand click on the 'Manuscripts with Decisions' queue in the Author Area.

The overall evaluation is very positive and we would like to publish a revised manuscript in Development that addresses the comments of the referees. If you do not agree with any of their criticisms or suggestions explain clearly why this is so.

We are aware that you may currently be unable to access the lab to undertake experimental revisions. If it would be helpful, we encourage you to contact us to discuss your revision in greater detail. Please send us a point-by-point response indicating where you are able to address concerns raised (either experimentally or by changes to the text) and where you will not be able to do so within the normal timeframe of a revision. We will then provide further guidance. Please also note that we are happy to extend revision timeframes as necessary.

Reviewer 1

Advance summary and potential significance to field

Much progress has been made in recent years in understanding the role of LLR-containing receptors in translating the segmentation gene code into planar polarized protein localizations and cell behaviors during Drosophila germband extension. This manuscript is an important addition to these studies.

© 2022. Published by The Company of Biologists under the terms of the Creative Commons Attribution License (https://creativecommons.org/licenses/by/4.0/).

The first part describing an in silico screen for genes with striped expression patterns under pair-rule control provides useful confirmation of previous studies and also identifies candidate genes for future studies (in addition to those which are further investigated in this manuscript).
The authors confirm a role for Tartan in maintaining parasegment boundaries during germband extension under pair-rule control, but also further show that Toll-2 contributes to maintenance of parasegment boundaries during extended germband stages under Wingless control. They then go on describe some novel genetic tools and computational pipelines for live tracking of parasegmental boundaries, and use these to more accurately quantify the tartan phenotype, showing that loss of tartan severely affects parasegment boundary straightness from the start of germband extension onwards but that straightness starts to return after about 40 minutes, consistent with Toll-2 starting to take over this function. Finally, the authors address the paradox that loss of tartan affects straightness of all parasegmental boundaries, but loss of its upstream regulator ftz only affects alternate boundaries. They use their new tools to rigorously confirm this result and also provide evidence that changes in Toll receptor expression in ftz mutants could be responsible.
Overall, this is a thoughtful and rigorous study. Although some aspects are confirmatory, I don't see this as a weakness, not only because conformation of results is important, but also because the use of new tools in this manuscript provides greater detail regarding previously reported phenotypes. I also note that the tools will also be of use to other groups.

Comments for the author

Minor comments:
line 102 "for for specifiying" – economically combines both a word duplication and (I think) a typo?
lines 185-186 – notwithstanding the failure to detect a change in Myo-II enrichment on PSBs in tartan mutants at extended germband stages, it seems worth commenting on that PSB straightness is still reduced. Possibly this could be due to the earlier defect in tartan mutants, rather than reflecting an ongoing requirement for tartan activity?
line 199 "strippy" – the definitions of this word on urbandictionary.com make for amusing reading, but I can't help thinking that none of them are quite appropriate here?
lines 327-328 "After 40 minutes into germband extension, tartan PSB straightness curves start to increase towards wildtype" – I'm not quite sure where the figure of 40 minutes comes from? Looking at e.g. Fig.5F both wt and trn curves start to rise at about 20 minutes into GBE. I could believe that at 40 minutes, trn starts to rise faster than wt, but this is quite subjective. Do the authors have a more objective criterion for making this judgment, or is it just this curve shape I should be looking at?
With regards to this "rescue" of boundary straightness, how straightforward would it be to look at e.g. tartan Toll-2 double mutants (at least in fixed tissue)? I think the prediction is that the rescue might not occur (if Toll-2 is responsible) and also the phenotype in extended germband stages will be greater than that for loss of Toll-2 alone. Such double mutants might also help to explain the retention of "odd" boundary straightness in ftz mutants.
line 796 "by stage 10, toll-2 expression is absent" – could the authors comment on the remaining labeling seen in green on the image? The periodicity doesn't seem to agree with the claim that there is no expression.

Reviewer 2

Advance summary and potential significance to field

Previous work in the field has shown that actomyosin cables form along parasegment boundaries and that these boundaries restrict cell movement between parasegments (Monier et al., 2010). While these boundary actomyosin cables were shown to depend on Wg signaling after germband extension, the cell surface receptors responsible for establishing and maintaining parasegmental actomyosin cables are unknown. Using a screen, the authors identified the cell surface receptor, Tartan, as being required to establish parasegmental boundaries during germband extension. Additionally, they demonstrated that maintenance of the boundaries is passed to a different cell surface receptor, Toll-2, a Wg target gene, after germband extension. Using a combination of transcriptional readouts and live imaging, the authors show that tartan mRNA expression borders parasegmental boundaries early in germband extension and retracts from the boundaries at the developmental time corresponding to the initiation of toll-2 expression at the boundaries.

© 2022. Published by The Company of Biologists under the terms of the Creative Commons Attribution License (https://creativecommons.org/licenses/by/4.0/).

Overall, the manuscript advances the field by thoroughly detailing the temporal evolution of cell surface receptor requirements that establish and then maintain the parasegmental boundary actomyosin cables in Drosophila; a unique and important contribution to the field.

- Adam C Martin

Comments for the author

1) One general comment is to discuss how cell intercalation affects the expression patterns of these genes during the observed time stages. E.g. Are two receptors needed because cell movement changes inter-relationship between cells?
2) Introduction: For the sake of completeness, it is worth pointing out that cell protrusion also plays a pivotal role in convergent extension in both invertebrate and vertebrate systems (Huebner and Wallingford 2018).
3) Figure 1c and Supplementary Figure 1 have some inverted, duplicated images. For the sake of transparency, I recommend directly stating that the images are the same between the two figures or using different images.
4) The diagram in Supplemental Figure 3, later used in Figure 2 are not immediately understandable.
Notably Supp Fig 3, is referenced first but this graphical style is not explained in-depth until later when Figure 2 is referenced. To improve clarity, I would advise explaining the how to interpret the figure the first time it is referenced, or if there is worry that a subset of readers will not engage the supplements, in both locations.
5) Line 170, fulfills – spelling
6) Figure 2: When describing index of straightness, reader understanding of the analysis could be improved by describing how the measurements were taken in a manner similar to the description given for measurement of straightness in Fig 4 and 5. To this point, it would also be useful to include a sample image of the Myosin II that was quantified for Fig 2D and G.
Also for the quantifications in Fig 2C, D, F, and G, information about the exact stages being used to making these measurements should be included. In a prior figure, there were subdivision with-in the categories of GBE and Extended Germ-Band. Are these measurements an average of the included stages, or a specific stage with-in that range. This seems particularly relevant for the GBE category during which there is a change in tartan and toll-2 expression as shown in Supplemental Figure 3.
7) For Figures 2B and E, phospo-Tyrosine staining appears to be used as a cell membrane marker, and adding this to the figure caption would help clarify the inclusion of the marker in the figure.

<u>Reviewer 3</u>

Advance summary and potential significance to field

In this manuscript, Sharrock, Blanchard, Evans, and Sanson use an unbiased screening approach to identify cell surface proteins involved in the formation of parasegmental boundaries (PSBs) in the early Drosophila embryo. Using a combination of in silico database mining and in situ validation using HCR, they identified 19 genes that encode membrane proteins expressed in striped patterns during stages 5-10 of development (including tartan, comm, comm2, and Toll-2,6,7,8). Analysis of boundary straightness in tartan mutants confirmed an earlier finding that tartan is required for myosin enrichment at PSBs and boundary straightness during germband extension. They found no such requirement for another candidate, comm. Interestingly, they found that boundary straightness eventually recovered to wild-type levels during extended germband stages in tartan mutants, indicating that another mechanism takes over the function of tartan at later stages. Based on the long-standing observation that PSBs are maintained by wingless signaling, they found that expression of Toll-2 was disrupted in wingless mutants, and Toll-2 expression is required for myosin enrichment at PSBs and boundary straightness specifically during the extended germband stages. The authors then use a new En-MS2 reporter and computational methods to describe the behavior of PSBs and nearby interfaces in wild-type vs. tartan mutants.

© 2022. Published by The Company of Biologists under the terms of the Creative Commons Attribution License (https://creativecommons.org/licenses/by/4.0/).

This manuscript uses sensitive quantitative methods to identify new Toll receptor functions and unexpected relationships between patterned cell surface proteins required for epithelial morphogenesis.
The experiments are carefully done and the authors use novel means of cell tracking and analysis (i.e., En-MS2 labeling of PSBs in live embryos, and boundary straightness plots over time). Although the finding that Tartan is required for polarity at PSBs during germband extension is not novel, the authors show for the first time that this requirement is transient, and they make a strong case that Toll-2 regulates boundary straightness downstream of wingless during extended germband stages. Considering the fact that Toll-2 stripes don't abut PSBs, but rather straddle them, this is an intriguing and non-intuitive finding that will certainly fuel future investigations. This manuscript is appropriate in principle for publication in Development, given the authors can address the following comments.

Comments for the author

1. Considering the importance of the Toll-2 findings to the overall paper, a more detailed analysis of cell shape changes in a Toll-2 mutant should be included, similar to the analysis of tartan mutants in Figure 5.
Does alignment at PSBs change over time in Toll-2 mutants, and are these changes the inverse of those seen in tartan mutants?
2. Images of myosin-II enrichment at PSBs in tartan, comm, and Toll-2 mutants should be shown to support the data in the plots.
3. The authors show that Toll-2 is required for myosin-II enrichment and boundary straightness at PSBs in the extended germ-band stage. They also show that Toll-2 expression straddles the PSB at this stage. If the authors break down the data in Fig. 3E, do they observe effects of loss of Toll-2 at both the PSB and PSB+1 borders at the GBE and extended germ-band stages, or only at the PSB? Are there differences in PSB straightness at odd versus even-numbered PSBs in Toll-2 mutants, or are the defects similar at all PSBs?
4. In Figure 1A, the number of candidates that meet the criteria in each of the Venn diagram circles should be indicated. This will clarify that the 822 genes expressed in AP stripes in at least one of the four databases do not necessarily represent the only candidates with striped expression at this stage, as not all Drosophila genes have available expression data.
5. In Figure 1A, the authors should explain the criteria used to conclude that genes in the bottom circle are pair-rule regulated, as some of this evidence may be indirect.
6. It would be helpful to include a schematic of the Toll-2 expression pattern in a wg mutant during GBE and extended germ-band stages in Figure 3.
7. The section on ftz dsRNA-injected embryos was difficult to follow. As a primary pair-rule gene, loss of ftz disrupts the expression of many genes, including other pair-rule genes, which could produce non-intuitive results. It might help to provide more of a rationale for this experiment at the beginning of this paragraph to make it easier for readers to interpret the different comparisons.
8. Scale bars are missing in some figures.
9. Other comments
The authors should indicate that the measurements in Fig. 2D, 2G, and 3E are on a log2 scale.
Is the blue wg expression data missing from Figure 3B? A positive control should be added.
The molecular nature of the tartan, comm, wg, Toll-2 and ftz alleles should be explained in the methods to justify that these are null alleles.
The term cell surface receptors is not quite accurate throughout the paper, since comm and comm2 are not cell surface receptors.
"Toll-like" should be changed to "Toll" or "Toll-related" as "Toll-like" generally refers to vertebrate Toll family members.
line 102: for is repeated twice
lines 127, 151, 338: stripy should be striped
line 199: strippy should be striped
line 477: error in boundaries
line 505: increase should be increases
line 764: interfaces should be interface

© 2022. Published by The Company of Biologists under the terms of the Creative Commons Attribution License (https://creativecommons.org/licenses/by/4.0/).

First revision

Author response to reviewers' comments

Summary of changes in the revised version:

We have updated Figures 2, 3 and Sup. Fig. 8 and consolidated the manuscript in two mains areas:

1) Toll-2 requirement for parasegmental boundary maintenance:
We wanted to confirm this requirement with another mutant allele of *toll-2*. While constructing new strains bearing *sqh-GFP* combined with different mutant alleles, we realised that a mistake had been made with the mutant allele used in the first version of the manuscript. While we thought we had analysed *sqh-GFP[KI]; Toll2[k02701]/CyO, wg-lacZ* , we had not realised that this *toll-2* allele expresses *lacZ* at a significant level and this pattern got mistaken for the pattern in *CyO, wg-lacZ* during strain building (*wg-lacZ* and *toll2-lacZ* segmentally-repeated patterns are very similar). So the stock is incorrect and the selection against *lacZ* has not identified reliably the *toll-2* homozygous embryos (in fact it is likely the *CyO* homozygous embryos have been analysed instead). So we have removed the corresponding data from the manuscript and repeated the experiment with a different allele of *toll-2, Toll-2[D7-35]*, having built the strain *sqh-GFP[KI]; toll-2[D7-35]/CyO, eve-lacZ*. We do not find a requirement for *toll-2* in this new experiment, see new data in **Fig. 3D**. To have a positive control, we also built a strain *sqh- GFP[KI]; wg[CX4], en-LacZ/CyO* and performed the same quantifications (*en-lacZ* is used to find the PSBs in absence of Wg signalling). As expected from our previous published work, actomyosin enrichment and straightness are decreased at PSBs in absence of Wg signalling during germband extended stages (but not during germband extension) (new **Fig. 3E**). This also confirms that our quantification of native fluorescence from *sqh-GFP* is sound (we had quantified P-Sqh in previous publications but for this study we have developed a method not dependent upon antibody staining). We have also improved our normalisation methods for quantifying the *sqh-GFP* signal at boundaries (in updated Methods), and present updated plots for the quantifications in *tartan* (**Fig. 2E,F**) and *comm* (**Fig. 2H,I**) mutants. The improvement in normalisation means the quantifications are more sensitive and we now detect a small decrease in actomyosin enrichment in *comm* mutants during GBE. This will have to be explored further in future. We have updated the manuscript throughout, including the title, to reflect the above corrections.

2) Investigating why odd-numbered PSBs are functional in *ftz* mutants:
This result was puzzling because *tartan*'s expression had been reported to be lost in *ftz* mutants (Chang et al 1993), so why then did the odd-numbered PSBs in *ftz* mutants behave as WT in our assay of boundary straightness in live embryos? We had proposed that other receptors might have their expression changed in *ftz* mutants and perhaps rescue boundary function. We have now investigated this further by repeating HCR experiments in *ftz* mutants embryos for all the receptors expressed near PSBs found in our unbiased screen, including *tartan* (summarised in updated **Sup. Fig. 8A,D**). We find that while the expression of many receptors do change in *ftz* mutants in the region corresponding to the *ftz* domain, their expression is unchanged at odd-numbered PSBs. Moreover, we find that *tartan* is detectably expressed at odd-numbered PSBs (while it is gone at the vicinity of even-numbered PSBs as expected). We quantify this remaining expression and show that it is comparable to the level of expression at odd-numbered PSB in wildtype embryos and that there is also a sharp differential at the boundary. We conclude that the simplest explanation for why odd-numbered boundary function as wild-type in *ftz* mutants is that there is sufficient expression of *tartan* left along this boundary in *ftz* mutants (likely under the control of other pair-rule inputs). We characterize this remaining expression of *tartan* in an updated **Sup. Fig. 8B,C,E,F**. Note that we have attempted to build a *tartan ftz* double mutant to demonstrate that *tartan* is indeed responsible for odd-numbered PSB function in *ftz* mutants, but did not recover recombinants in time for this revision (*tartan* and *ftz* are close, separated by 8cM).

Note that abstract and discussion have been shortened to comply with Development's word limits.

Point-by-point response to the referees' comments:

Reviewer 1 Advance Summary and Potential Significance to Field:
Much progress has been made in recent years in understanding the role of LLR-containing

© 2022. Published by The Company of Biologists under the terms of the Creative Commons Attribution License (https://creativecommons.org/licenses/by/4.0/).

receptors in translating the segmentation gene code into planar polarized protein localizations and cell behaviors during Drosophila germband extension. This manuscript is an important addition to these studies.
The first part describing an in silico screen for genes with striped expression patterns under pair-rule control provides useful confirmation of previous studies and also identifies candidate genes for future studies (in addition to those which are further investigated in this manuscript).
The authors confirm a role for Tartan in maintaining parasegment boundaries during germband extension under pair-rule control, but also further show that Toll-2 contributes to maintenance of parasegment boundaries during extended germband stages under Wingless control. They then go on describe some novel genetic tools and computational pipelines for live tracking of parasegmental boundaries, and use these to more accurately quantify the tartan phenotype, showing that loss of tartan severely affects parasegment boundary straightness from the start of germband extension onwards but that straightness starts to return after about 40 minutes, consistent with Toll-2 starting to take over this function. Finally, the authors address the paradox that loss of tartan affects straightness of all parasegmental boundaries, but loss of its upstream regulator ftz only affects alternate boundaries. They use their new tools to rigorously confirm this result and also provide evidence that changes in Toll receptor expression in ftz mutants could be responsible.
Overall, this is a thoughtful and rigorous study. Although some aspects are confirmatory, I don't see this as a weakness, not only because conformation of results is important, but also because the use of new tools in this manuscript provides greater detail regarding previously reported phenotypes. I also note that the tools will also be of use to other groups.

Reviewer 1 Comments for the Author:
Minor comments:
line 102 "for for specifiying" – economically combines both a word duplication and (I think) a typo?
Now corrected.

lines 185-186 – notwithstanding the failure to detect a change in Myo-II enrichment on PSBs in tartan mutants at extended germband stages, it seems worth commenting on that PSB straightness is still reduced. Possibly this could be due to the earlier defect in tartan mutants, rather than reflecting an ongoing requirement for tartan activity?
Indeed the straightness is statistically reduced at extended germband stages (Fig. 2 C), but this reduction is much smaller than at the other two time-points in the graph. This decreasing requirement of *tartan* in the course of development matches the straightness analysis in live embryos (Fig. 5D). Overall, we find that straightness is a more sensitive measure of boundary function than actomyosin enrichment, so it could be that while we cannot detect a difference in actomyosin enrichment, we still detect a defect in boundary straightness. We also agree with the referee that it could be that the lack of *tartan* is still having an impact on boundary straightness from a requirement in actomyosin enrichment at earlier stages.

line 199 "strippy" – the definitions of this word on urbandictionary.com make for amusing reading, but I can't help thinking that none of them are quite appropriate here?
© - replaced by "striped" throughout manuscript and figures.

lines 327-328 "After 40 minutes into germband extension, tartan PSB straightness curves start to increase towards wildtype" – I'm not quite sure where the figure of 40 minutes comes from? Looking at e.g. Fig.5F, both wt and trn curves start to rise at about 20 minutes into GBE. I could believe that at 40 minutes, trn starts to rise faster than wt, but this is quite subjective. Do the authors have a more objective criterion for making this judgment, or is it just this curve shape I should be looking at?

We are just looking at the curve shape. We agree with the referee that PSB curves both become straighter from 20 minutes in WT and *tartan* mutants. This increase is parallel until about 40 minutes, when straightness in *tartan* increases more quickly. We could formally compare rate of changes, but perhaps a qualitative description is sufficient here. The fact that in *tartan*, the PSB curve starts straightening again from 20 minutes, as for the wild- type PSB and in contrast with the -1 and +1 curves, suggests that actomyosin is getting re-enriched as early as 20 minutes into GBE. This is consistent with *tartan* acting very early in germband extension. We have added more emphasis about this in the results and discussion.

© 2022. Published by The Company of Biologists under the terms of the Creative Commons Attribution License (https://creativecommons.org/licenses/by/4.0/).

With regards to this “rescue” of boundary straightness, how straightforward would it be to look at e.g. tartan Toll-2 double mutants (at least in fixed tissue)? I think the prediction is that the rescue might not occur (if Toll-2 is responsible) and also the phenotype in extended germband stages will be greater than that for loss of Toll-2 alone. Such double mutants might also help to explain the retention of “odd” boundary straightness in ftz mutants.

For this revision, we focused on consolidating the Toll-2 result, which led to the correction described in the revision summary. Our updated results do not detect a role for *toll-2* on its own, but we agree with the reviewer that a role of *toll-2* might be revealed in a *tartan toll2* double mutant. This is beyond this revision, but we will consider to do this experiment in future.

line 796 “by stage 10, toll-2 expression is absent” – could the authors comment on the remaining labeling seen in green on the image? The periodicity doesn’t seem to agree with the claim that there is no expression.
We have revised Figure 3, including improving the presentation of the HCR images based on the reviewer comment. In a *wg* mutant, we find that *toll-2* segmental expression fades away with similar timings as *slp1* expression (Fig. 3B). In addition to a clear segmental pattern, *toll-2* is expressed at a weaker level in the midline and around forming tracheal pits in extended germband stages. In a *wg* mutant, this weak expression is unchanged and is at comparable level to the disappearing segmental toll-2 expression, which is why it becomes noticeable in the images of stage 10 (and to a lesser extent stage 9).

Reviewer 2 Advance Summary and Potential Significance to Field:
Previous work in the field has shown that actomyosin cables form along parasegment boundaries and that these boundaries restrict cell movement between parasegments (Monier et al., 2010). While these boundary actomyosin cables were shown to depend on Wg signaling after germband extension, the cell surface receptors responsible for establishing and maintaining parasegmental actomyosin cables are unknown. Using a screen, the authors identified the cell surface receptor, Tartan, as being required to establish parasegmental boundaries during germband extension. Additionally, they demonstrated that maintenance of the boundaries is passed to a different cell surface receptor, Toll-2, a Wg target gene, after germband extension. Using a combination of transcriptional readouts and live imaging, the authors show that tartan mRNA expression borders parasegmental boundaries early in germband extension and retracts from the boundaries at the developmental time corresponding to the initiation of toll-2 expression at the boundaries.

Overall, the manuscript advances the field by thoroughly detailing the temporal evolution of cell surface receptor requirements that establish and then maintain the parasegmental boundary actomyosin cables in Drosophila; a unique and important contribution to the field.

- Adam C Martin

Reviewer 2 Comments for the Author:

1) One general comment is to discuss how cell intercalation affects the expression patterns of these genes during the observed time stages. E.g. Are two receptors needed because cell movement changes inter- relationship between cells?
Cell intercalation in GBE will double, in average, the number of cells along AP. In our previous study (Tetley et al 2016), we have found that when cells intercalate, actomyosin enrichment is lost between new neighbours with the same cell AP identity/ original AP position. Based on this we think that boundary formation is a direct consequence of heterophilic interactions between cells. When cells intercalate, cells move away from the boundary, and we think boundary integrity is maintained because cell-cell interfaces pull on each other and align, correcting any disturbances. As for why there would be more than one receptor - tartan, as Toll-2,6,8 are regulated by the pair-rule genes, which is the gene regulatory network relevant for GBE. But beyond GBE, pair- rule influence disappears, so other regulatory inputs must take over at PSBs, one of which is Wg signalling.

2) Introduction: For the sake of completeness, it is worth pointing out that cell protrusion also plays a pivotal role in convergent extension in both invertebrate and vertebrate systems (Huebner and Wallingford, 2018).

© 2022. Published by The Company of Biologists under the terms of the Creative Commons Attribution License (https://creativecommons.org/licenses/by/4.0/).

Since our focus is boundary formation, we only give a brief introduction on convergence extension.

3) Figure 1c and Supplementary Figure 1 have some inverted, duplicated images. For the sake of transparency, I recommend directly stating that the images are the same between the two figures or using different images.
Yes, Fig 1C is intended as an illustration of the approach and we chose to show a subset of the key candidate HCR as examples. We now specify in the legend of figure 1 that the data presented in C is the same data as in supp fig 1.

4) The diagram in Supplemental Figure 3, later used in Figure 2 are not immediately understandable.
Notably Supp Fig 3, is referenced first but this graphical style is not explained in-depth until later when Figure 2 is referenced. To improve clarity, I would advise explaining the how to interpret the figure the first time it is referenced, or if there is worry that a subset of readers will not engage the supplements, in both locations.

We have now added a couple of sentences to introduce the diagrams at the location suggested by the referee. In particular, we explain that the representative parasegments are divided in 4 domains to help the mapping. Although these domains correspond approximately to the number of cells along AP parasegment at the start of GBE (3.7 cells as measured in our study Tetley et al 2016), then the number of cells increase steadily as polarised cell intercalation proceeds during GBE. Cell number increases further during extended germband stages when cell starts dividing. We decided that trying to map patterns to cell numbers was too complex, hence the representation in 4 domains, which has the advantage to be consistent throughout development (see Sup. Fig. 3). We have also added a new section in the Methods called “Embryo staging and mapping of expression patterns by HCR”

5) Line 170, fulfills – spelling
We are using the UK spelling with only one l.

6) Figure 2: When describing index of straightness, reader understanding of the analysis could be improved by describing how the measurements were taken in a manner similar to the description given for measurement of straightness in Fig 4 and 5. To this point, it would also be useful to include a sample image of the Myosin II that was quantified for Fig 2D and G.

We now include images in Figure 2 (new panels B. C, C’) to explain how we quantify boundary straightness and Sqh-GFP native fluorescence in fixed samples. We have also added a section in the Methods “Quantification of Myosin II intensities and boundary straightness at PSBs”, which was missing.

Also for the quantifications in Fig 2C, D, F, and G, information about the exact stages being used to making these measurements should be included. In a prior figure, there were subdivision with-in the categories of GBE and Extended Germband. Are these measurements an average of the included stages, or a specific stage with-in that range. This seems particularly relevant for the GBE category during which there is a change in tartan and toll-2 expression as shown in Supplemental Figure 3.
Figure 2 and 3, “early GBE” corresponds to stage 7 embryos, “late GBE”, stage 8 and “Extended germband”, stage 9 and 10. We have clarified this staging in the legends and also in the new section of the Methods mentioned above.

7) For Figures 2B and E, phospo-Tyrosine staining appears to be used as a cell membrane marker, and adding this to the figure caption would help clarify the inclusion of the marker in the figure.
This is correct – we used a Phospho-tyrosine antibody, which labels the adherens junctions in Drosophila, to label the cell membranes in fixed samples in order to identify precisely the junctions corresponding to PSBs. We now show a new image in Figure 2 (panel B) showing the pTyr staining and added clarifications in the text.

© 2022. Published by The Company of Biologists under the terms of the Creative Commons Attribution License (https://creativecommons.org/licenses/by/4.0/).

Reviewer 3 Advance Summary and Potential Significance to Field:
In this manuscript, Sharrock, Blanchard, Evans, and Sanson use an unbiased screening approach to identify cell surface proteins involved in the formation of parasegmental boundaries (PSBs) in the early Drosophila embryo. Using a combination of in silico database mining and in situ validation using HCR, they identified 19 genes that encode membrane proteins expressed in striped patterns during stages 5-10 of development (including tartan, comm, comm2, and Toll-2,6,7,8). Analysis of boundary straightness in tartan mutants confirmed an earlier finding that tartan is required for myosin enrichment at PSBs and boundary straightness during germband extension. They found no such requirement for another candidate, comm. Interestingly, they found that boundary straightness eventually recovered to wild-type levels during extended germband stages in tartan mutants, indicating that another mechanism takes over the function of tartan at later stages. Based on the long-standing observation that PSBs are maintained by wingless signaling, they found that expression of Toll-2 was disrupted in wingless mutants, and Toll-2 expression is required for myosin enrichment at PSBs and boundary straightness specifically during the extended germband stages. The authors then use a new En-MS2 reporter and computational methods to describe the behavior of PSBs and nearby interfaces in wild-type vs. tartan mutants.

This manuscript uses sensitive quantitative methods to identify new Toll receptor functions and unexpected relationships between patterned cell surface proteins required for epithelial morphogenesis.
The experiments are carefully done and the authors use novel means of cell tracking and analysis (i.e., En- MS2 labeling of PSBs in live embryos, and boundary straightness plots over time). Although the finding that Tartan is required for polarity at PSBs during germband extension is not novel, the authors show for the first time that this requirement is transient, and they make a strong case that Toll-2 regulates boundary straightness downstream of wingless during extended germband stages. Considering the fact that Toll-2 stripes don't abut PSBs, but rather straddle them, this is an intriguing and non-intuitive finding that will certainly fuel future investigations. This manuscript is appropriate in principle for publication in Development, given the authors can address the following comments.

Reviewer 3 Comments for the Author:
1. Considering the importance of the Toll-2 findings to the overall paper, a more detailed analysis of cell shape changes in a Toll-2 mutant should be included, similar to the analysis of tartan mutants in Figure 5. Does alignment at PSBs change over time in Toll-2 mutants, and are these changes the inverse of those seen in tartan mutants?

As presented in the revision summary, we have now corrected our initial report and cannot detect a requirement for *toll-2* in fixed samples. In future, we will consider analysing a double mutant *tartan toll-2* as suggested by reviewer 1. However, one obstacle is that the MS2/MCP system we have built does not mark substantially the *engrailed* stripes at germband extended stages. This is because the expression reported from the Engrailed enhancer region we used fades away at the end of germband extension. So we will have to build new tools to follow boundary formation at germband extended stages.

2. Images of myosin-II enrichment at PSBs in tartan, comm, and Toll-2 mutants should be shown to support the data in the plots.
This was also requested by reviewer 1. We know show example images in Figure 2 to explain how we quantify boundary straightness and Sqh-GFP native fluorescence in fixed samples. We provide an example for the wildtype, but not for the mutants because of limitation of space.

3. The authors show that Toll-2 is required for myosin-II enrichment and boundary straightness at PSBs in the extended germband stage. They also show that Toll-2 expression straddles the PSB at this stage. If the authors break down the data in Fig. 3E, do they observe effects of loss of Toll-2 at both the PSB and PSB+1 borders at the GBE and extended germband stages, or only at the PSB? Are there differences in PSB straightness at odd versus even-numbered PSBs in Toll-2 mutants, or are the defects similar at all PSBs?
We have now corrected our Toll-2 results, as indicated in the summary. In Figure 2D, we detect an increase of the ratio PSB/+1 in late GBE, which is consistent with a decrease in +1 interfaces in *toll-2* mutants. This is expected based on findings by Pare et al 2019 and Lavalou et al 2021. We

© 2022. Published by The Company of Biologists under the terms of the Creative Commons Attribution License (https://creativecommons.org/licenses/by/4.0/).

now comment on this in the legend. This is also consistent with the pattern of expression of *toll-2*, which straddles the PSBs rather than bordering them.

4. In Figure 1A, the number of candidates that meet the criteria in each of the Venn diagram circles should be indicated. This will clarify that the 822 genes expressed in AP stripes in at least one of the four databases do not necessarily represent the only candidates with striped expression at this stage, as not all Drosophila genes have available expression data.
Our aim for Figure 1 is to provide an overview of the screen, and we'd rather not to crowd it by adding numbers. Gene numbers are given in the Results text and there is also more detailled information about the screen in the Methods.

5. In Figure 1A, the authors should explain the criteria used to conclude that genes in the bottom circle are pair-rule regulated, as some of this evidence may be indirect.
More detailed information about the screen is given in the Methods.

6. It would be helpful to include a schematic of the Toll-2 expression pattern in a wg mutant during GBE and extended germband stages in Figure 3.
We have now improved Figure 3. Because of constrains of space we haven't added a schematic, but we hope that the figures now show more clearly that *toll-2* segmental expression fades away in *wg* mutant, similarly to the *slp1* control, which is also regulated by Wingless signalling.

7. The section on ftz dsRNA-injected embryos was difficult to follow. As a primary pair-rule gene, loss of ftz disrupts the expression of many genes, including other pair-rule genes, which could produce non- intuitive results. It might help to provide more of a rationale for this experiment at the beginning of this paragraph to make it easier for readers to interpret the different comparisons.
We have now rewritten the *ftz* section, incorporating our new results and providing more rationale for the experiments - we hope that it is easier to follow.

8. Scale bars are missing in some figures.
Missing scale bars have now been added to images of fixed embryos.

9. Other comments

The authors should indicate that the measurements in Fig. 2D, 2G, and 3E are on a log2 scale.
The measurements are on a log(10) scale and we have now labelled the axes accordingly in graphs in Figure 2 and 3.

Is the blue wg expression data missing from Figure 3B? A positive control should be added.
We have improved the presentation of the HCR data in Figure 3 and removed the Wg channel as it was not useful. Our control is *slp1*, which is known to require Wg signalling for the maintenance of its transcription.

The molecular nature of the tartan, comm, wg, Toll-2 and ftz alleles should be explained in the methods to justify that these are null alleles.
Molecular information, when it exists, is given in Flybase. We have added in the Methods some more information about the alleles we used.

The term cell surface receptors is not quite accurate throughout the paper, since comm and comm2 are not cell surface receptors.
We have now checked throughout the manuscript that we mentioned that Comm and Comm2 are regulators of cell surface receptors, rather than cell receptors themselves.

"Toll-like" should be changed to "Toll" or "Toll-related" as "Toll-like" generally refers to vertebrate Toll family members.

Both nomenclatures are found in *Drosophila* and vertebrate literature. We decided to be consistent with Flybase as it uses the term "Toll-like" in its description of the corresponding genes (see for example http://flybase.org/reports/FBgn0287775)

© 2022. Published by The Company of Biologists under the terms of the Creative Commons Attribution License (https://creativecommons.org/licenses/by/4.0/).

line 102: for is repeated twice
Corrected
lines 127, 151, 338: stripy should be striped
corrected
line 199: strippy should be striped
corrected
line 477: error in boundaries
corrected
line 505: increase should be increases
corrected
line 764: interfaces should be interface
corrected

Second decision letter

MS ID#: DEVELOP/2021/200292

MS TITLE: Different temporal requirements for tartan and wingless in the formation of contractile interfaces at compartmental boundaries.

AUTHORS: Thomas E Sharrock, Jenny Evans, Guy B Blanchard, and Benedicte Sanson

I apologize for the delay before coming back to you. Ihave now received all the referees reports on the above manuscript, and have reached a decision. The referees' comments are appended below, or you can access them online: please go to BenchPressand click on the 'Manuscripts with Decisions' queue in the Author Area.

The overall evaluation is very positive and I am happy to tell you that we will accept publication of your manuscript following a few text edits, specifically the 3 comments of Reviewer 2 and point 4 of Reviewer 3.

Reviewer 1

Advance summary and potential significance to field

The manuscript has been thoughtfully revised. The story has changed a little, but this is a good thing, as an understandable error has been corrected, and also more data investigating the role of Tartan/Ftz in PSB formation has been added. I'm happy to recommend acceptance.

Comments for the author

Line 35: maybe should read 'partitioning of groups of cells'?
Line 398: maybe should read 'The above results suggest'?
Line 408: maybe should read 'at every PSB during'?

Reviewer 2

Advance summary and potential significance to field

Previous work in the field has shown that actomyosin cables form along parasegment boundaries and that these boundaries restrict cell movement between parasegments (Monier et al., 2010). While these boundary actomyosin cables were shown to depend on Wg signaling after germband extension, the cell surface receptors responsible for establishing and maintaining parasegmental actomyosin cables are unknown. Using a screen, the authors identified the cell surface receptor, Tartan, as being required to establish parasegmental boundaries during germband extension. Using

© 2022. Published by The Company of Biologists under the terms of the Creative Commons Attribution License (https://creativecommons.org/licenses/by/4.0/).

a combination of transcriptional readouts and live imaging, the authors show that tartan mRNA expression borders parasegmental boundaries early in germband extension. They showed that this requirement is transient and that a Wg-dependent mechanism operates after germband extension. Overall, the manuscript advances the field by thoroughly detailing the temporal evolution of cell surface receptor requirements that establish and then maintain the parasegmental boundary actomyosin cables in Drosophila; this study will be an important jumping-off point for future studies and provides useful tools to study the evolution of boundary patterns over the course of embryo development.

Comments for the author

The authors have addressed my points from the first revision. A couple of minor comments are as follows:
1) Line 166: ‘summarise genes expression patterns’ --> ‘summarise gene expression patterns’
2) Line 216: ‘temporally’ --> ‘temporarily’? otherwise ‘temporally’ and ‘after’ are redundant.
3) Line 616: The Martin et al., Nature, 2009 reference is incorrect. The Gap43mCherry flies, were published first in Martin et al., JCB, 2010.

Reviewer 3

Advance summary and potential significance to field

In this revised manuscript, the authors now show that Toll-2 does not regulate PSB straightness using a different Toll-2 allele, and they demonstrate that comm is required for interfacial contractility at PSBs using improved methods. The authors also add data showing that differences in tartan expression are retained at odd-numbered PSBs in ftz mutants, explaining why these these PSBs are still functional. Overall, this comprehensive study uses sensitive imaging and quantitative methods to demonstrate that the effect of Tartan at PSBs is transient and to narrow the list of candidates that could take over this essential function at later stages.

Comments for the author

The authors may want to consider the following suggestions.
1. As this study identifies comm as a new component regulating PSBs, the authors should include images of the comm myosin phenotype and a corresponding control.
2. "Pair-rule gene regulated" could be changed to "Predicted pair-rule gene regulated" or "Predicted pair-rule regulated" in Figure 1A, as the datasets mined do not contain proven pair-rule regulated genes.
3. The authors should clarify if wg regulates Toll-2 expression non-autonomously, ideally with a schematic of the wg and Toll-2 expression patterns. Can the authors indicate which cells express Toll-2 in Arm-Gal4/UAS-Wg embryos and speculate as to why Toll-2 is still expressed in stripes?
4. The molecular nature of the null alleles on lines 605-615 should be described and the comm references (if published) should be provided.
5. Other comments
On lines 150-151, the authors write that Toll-8 mRNA expression borders the PSBs at some point between stages 5 and 10. From Figure S3 the same could be said of Toll-6.
The authors should capitalize the first letter of Toll-6, etc. when referring to the gene names.
Lines 353-355 - "Tartan is the only receptor cue required" could be changed to "Tartan is the only known patterned receptor required"

© 2022. Published by The Company of Biologists under the terms of the Creative Commons Attribution License (https://creativecommons.org/licenses/by/4.0/).

Second revision

Author response to reviewers' comments

Reviewer 1 Advance Summary and Potential Significance to Field:
The manuscript has been thoughtfully revised. The story has changed a little, but this is a good thing, as an understandable error has been corrected, and also more data investigating the role of Tartan/Ftz in PSB formation has been added. I'm happy to recommend acceptance.

Reviewer 1 Comments for the Author:
Line 35: maybe should read 'partitioning of groups of cells'?
Corrected

Line 398: maybe should read 'The above results suggest'?
Corrected

Line 408: maybe should read 'at every PSB during'?
Corrected

Reviewer 2 Advance Summary and Potential Significance to Field:
Previous work in the field has shown that actomyosin cables form along parasegment boundaries and that these boundaries restrict cell movement between parasegments (Monier et al., 2010). While these boundary actomyosin cables were shown to depend on Wg signaling after germband extension, the cell surface receptors responsible for establishing and maintaining parasegmental actomyosin cables are unknown. Using a screen, the authors identified the cell surface receptor, Tartan, as being required to establish parasegmental boundaries during germband extension. Using a combination of transcriptional readouts and live imaging, the authors show that tartan mRNA expression borders parasegmental boundaries early in germband extension. They showed that this requirement is transient and that a Wg-dependent mechanism operates after germband extension. Overall, the manuscript advances the field by thoroughly detailing the temporal evolution of cell surface receptor requirements that establish and then maintain the parasegmental boundary actomyosin cables in Drosophila; this study will be an important jumping-off point for future studies and provides useful tools to study the evolution of boundary patterns over the course of embryo development.

Reviewer 2 Comments for the Author:
The authors have addressed my points from the first revision. A couple of minor comments are as follows:

1) Line 166: 'summarise genes expression patterns' --> 'summarise gene expression patterns'
Corrected

2) Line 216: 'temporally' --> 'temporarily'? otherwise 'temporally' and 'after' are redundant.
Corrected

3) Line 616: The Martin et al., Nature, 2009 reference is incorrect. The Gap43mCherry flies, were published
first in Martin et al., JCB, 2010.

Corrected.

Reviewer 3 Advance Summary and Potential Significance to Field:
In this revised manuscript, the authors now show that Toll-2 does not regulate PSB straightness using a different Toll-2 allele, and they demonstrate that comm is required for interfacial contractility at PSBs using improved methods. The authors also add data showing that differences in tartan expression are retained at odd-numbered PSBs in ftz mutants, explaining why these these PSBs are still functional. Overall, this comprehensive study uses sensitive imaging and quantitative methods to demonstrate that the effect of Tartan at PSBs is transient and to narrow the list of candidates that could take over this essential function at later stages.

© 2022. Published by The Company of Biologists under the terms of the Creative Commons Attribution License (https://creativecommons.org/licenses/by/4.0/).

Reviewer 3 Comments for the Author:
The authors may want to consider the following suggestions.

1. As this study identifies comm as a new component regulating PSBs, the authors should include images of the comm myosin phenotype and a corresponding control.
As explained in the rebuttal, we decided to show Myosin II images to explain our quantification method, rather than showing individual images of phenotypes.

2. "Pair-rule gene regulated" could be changed to "Predicted pair-rule gene regulated" or "Predicted pair-rule regulated" in Figure 1A, as the datasets mined do not contain proven pair-rule regulated genes.
The screen is based on predictions for all three criteria used so we don't think it is necessary to specify it, as it is implicit in the approach taken.

3. The authors should clarify if wg regulates Toll-2 expression non-autonomously, ideally with a schematic of the wg and Toll-2 expression patterns. Can the authors indicate which cells express Toll-2 in Arm-Gal4/UAS-Wg embryos and speculate as to why Toll-2 is still expressed in stripes?
We expect that Wg, a secreted ligand, would regulate Toll-2 non-autonomously but we do not demonstrate it here. What we can say is that Toll-2 behaves like Engrailed, a known target of Wingless in the embryo. When Wg is expressed everywhere, Engrailed expression broadens to fill half of the parasegment, no more. Toll-2 has the same behaviour. This is mentioned in line 237 and we have tweaked the sentence to make it clearer in response to the referee.

4. The molecular nature of the null alleles on lines 605-615 should be described and the comm references (if published) should be provided.
We have already indicated that all alleles used are genetic nulls, which is what is relevant in our study to test for a requirement. Note that alleles can be demonstrated to be genetically null without the molecular lesion being known. We had also indicated the additional alleles used to perform complementation tests. For comm, we have contacted Guy Tear to clarify the origin and molecular lesion of the allele used for quantifications and have added this information and a reference.

5. Other comments

On lines 150-151, the authors write that Toll-8 mRNA expression borders the PSBs at some point between stages 5 and 10. From Figure S3 the same could be said of Toll-6.
Added toll-6 to the list.

The authors should capitalize the first letter of Toll-6, etc. when referring to the gene names.
We are trying to follow the Drosophila nomenclature, where gene names are in italics and protein names are capitalized.

Lines 353-355 - "Tartan is the only receptor cue required" could be changed to "Tartan is the only known patterned receptor required"
Changed to "Tartan is the only patterned receptor required for actomyosin enrichment and straightness of PSBs at the start of GBE"

© 2022. Published by The Company of Biologists under the terms of the Creative Commons Attribution License (https://creativecommons.org/licenses/by/4.0/).

Third decision letter

MS ID#: DEVELOP/2021/200292

MS TITLE: Different temporal requirements for tartan and wingless in the formation of contractile interfaces at compartmental boundaries.

AUTHORS: Thomas E Sharrock, Jenny Evans, Guy B Blanchard, and Benedicte Sanson
ARTICLE TYPE: Research Article

I am happy to tell you that your manuscript has been accepted for publication in Development, pending our standard ethics checks.

© 2022. Published by The Company of Biologists under the terms of the Creative Commons Attribution License (https://creativecommons.org/licenses/by/4.0/).
